# Hox11 expressing regional skeletal stem cells are progenitors for osteoblasts, chondrocytes and adipocytes throughout life

**DOI:** 10.1038/s41467-019-11100-4

**Published:** 2019-07-18

**Authors:** Kyriel M. Pineault, Jane Y. Song, Kenneth M. Kozloff, Daniel Lucas, Deneen M. Wellik

**Affiliations:** 1 Department of Cell & Regenerative Biology, University of Wisconsin-Madison, Madison, WI 53705 USA; 2 Cellular and Molecular Biology Program, University of Michigan, Ann Arbor, MI 48109-2200 USA; 3 Department of Orthopedic Surgery, University of Michigan, Ann Arbor, MI 48109-2200 USA; 4 Division of Experimental Hematology and Cancer Research, Cincinnati Children’s Medical Center, Cincinnati, OH 45229-2842 USA

**Keywords:** Cartilage development, Mesenchymal stem cells, Development, Bone development

## Abstract

Multipotent mesenchymal stromal cells (MSCs) are required for skeletal formation, maintenance, and repair throughout life; however, current models posit that postnatally arising long-lived adult MSCs replace transient embryonic progenitor populations. We previously reported exclusive expression and function of the embryonic patterning transcription factor, Hoxa11, in adult skeletal progenitor-enriched MSCs. Here, using a newly generated *Hoxa11-CreER*^*T2*^ lineage-tracing system, we show *Hoxa11*-lineage marked cells give rise to all skeletal lineages throughout the life of the animal and persist as MSCs. *Hoxa11* lineage-positive cells give rise to previously described progenitor-enriched MSC populations marked by *LepR-Cre* and *Osx-CreER*, placing them upstream of these populations. Our studies establish that Hox-expressing cells are skeletal stem cells that arise from the earliest stages of skeletal development and self-renew throughout the life of the animal.

## Abstract (web summary)

Prior evidence suggested mesenchymal stromal cells (MSCs) required for skeletal formation, maintenance, and repair arise postnatally. Here, the authors show that Hoxa11 lineage-marked cells give rise to all skeletal lineages from embryogenesis through adulthood and are upstream progenitors of LepR- and Osx-lineage MSCs

## Introduction

H*ox* genes play well-established roles in patterning the embryonic skeleton. *Hox1* through *Hox13* paralogs are expressed and function regionally along the anterior–posterior axis of the axial skeleton, with the *Hox9-Hox13* paralogs co-opted to also pattern along the proximal to distal axis of the appendicular skeleton. The *Hox11* paralogs, *Hoxa11, Hoxc11,* and *Hoxd11*, pattern the sacral region of the spine and the zeugopod region of the limb (radius/ulna and tibia/fibula)^1^. Loss of *Hoxa11* and *Hoxd11* function during development results in dramatic mis-patterning of the forelimb zeugopod skeleton^2^. In addition to complete loss-of-function phenotypes observed during development, compound mutants exhibit defects in skeletal growth during postnatal stages and in adult fracture repair^3–5^.

Despite clear genetic evidence for *Hox* function in the skeleton, Hox expression is excluded from all mature skeletal cell types at all stages, including chondrocytes and osteoblasts^3,5,6^. Embryonically, Hox11 expression is observed in the developing zeugopod perichondrium immediately adjacent to Sox9-positive chondrocytes and, as the skeleton begins to ossify, expression continues in the periosteum, adjacent to Osterix-positive pre-osteoblasts^6^. At postnatal and adult stages, Hox11-expressing cells remain in the outer periosteal stroma adjacent to the osteoblast layer, and are additionally observed in the bone marrow and along the endosteal (inner) bone surface^3,5^. Adult Hox11-expressing stromal cells from the bone marrow and periosteum are identified by antibodies that mark progenitor-enriched mesenchymal stem/stromal cell (MSC) populations including PDGFRα/CD51 and Leptin-Receptor (LepR) as well as by *Leptin Receptor-Cre* (*LepR-Cre*)^5,7,8^. In vitro*, Hox11* mutant mesenchymal stromal cells (MSCs) are unable to differentiate into chondrogenic and osteogenic lineages, supporting a function for *Hox11* genes in this population^5^.

Several previous lineage labeling models have reported labeling of progenitor-enriched, bone marrow MSC populations, however, with the exception of *Prx1-Cre*, which labels the entire limb lateral plate mesoderm^9^, inducible lineage reporters only mark only a minor proportion of a multipotent, self-renewing population from postnatal stages, and only when induced at postnatal stages. These models include *Osterix-CreER* (*Osx-CreER*)*, Sox9-CreER, Aggrecan-CreER, PthrP-CreER*, and *Gremlin1-CreER*^10–13^. The *LepR-Cre* lineage reporter, while not inducible, eventually marks the majority of the progenitor-enriched MSCs in the adult bone marrow^8,10^. Of note, this model does not display robust contribution to osteoblasts until 5–6 months of age^8,10^. Recent evidence showed embryonic and postnatal *Gli1-CreER* lineage marked cells are multi-potent and give rise to LepR-positive bone marrow MSCs in the adult^14^. However, the pattern of contribution to the skeleton differs significantly based on the induction time points, indicating that this lineage-marked population is not equivalent at embryonic and postnatal stages.

Previous work has genetically established the importance of *Hox11* genes in embryonic skeletal development, postnatal growth, and adult fracture repair^3–6^. Considering the continuity in Hoxa11eGFP expression in the zeugopod skeleton throughout life and the recent identification of adult, Hox11-expressing cells as skeletal MSCs, we sought to test the progenitor capacity of the Hox11-expressing population throughout the life of the animal. To do this, we generated a *Hoxa11-CreER*^*T2*^ lineage-tracing allele and we find that *Hoxa11*-lineage marked cells continuously give rise to all the skeletal mesenchymal lineages (cartilage, bone, and fat) during embryonic development, postnatal growth, at homeostasis and in response to injury. Even when lineage labeling is initiated at embryonic stages, *Hoxa11*-lineage marked stromal cells arising from this lineage co-express MSC markers PDGFRα/CD51 and LepR. In contrast to other reported embryonically induced progenitor populations, the *Hoxa11*-lineage is maintained as progenitor-enriched MSCs in adult bone marrow and demonstrate strong lineage labeling of all skeletal lineages through at least 1 year of age. Further, *Hoxa11* lineage-marked MSCs also express Hoxa11eGFP at all stages examined. These results provide strong evidence for the in vivo self-renewal of this MSC population.

To understand the lineage relationships between Hox11-expressing cells and other genetically marked progenitor/MSC populations, we compared Hoxa11eGFP expression to cells genetically lineage-labeled by *LepR-Cre* and *Osx-CreER*^8,10,11^. Herein, we show that Hox11-expressing cells serve as upstream progenitors that give rise to cells marked by these other genetic models. Taken together, these data support Hox-expressing skeletal, stromal cells as a bona fide skeletal stem cell population and demonstrates the presence of a specific, lineage-continuous skeletal stem cell population from embryonic stages throughout life.

## Results

### *Hox11* expression defines a continuous progenitor population

**Fig. 1:**
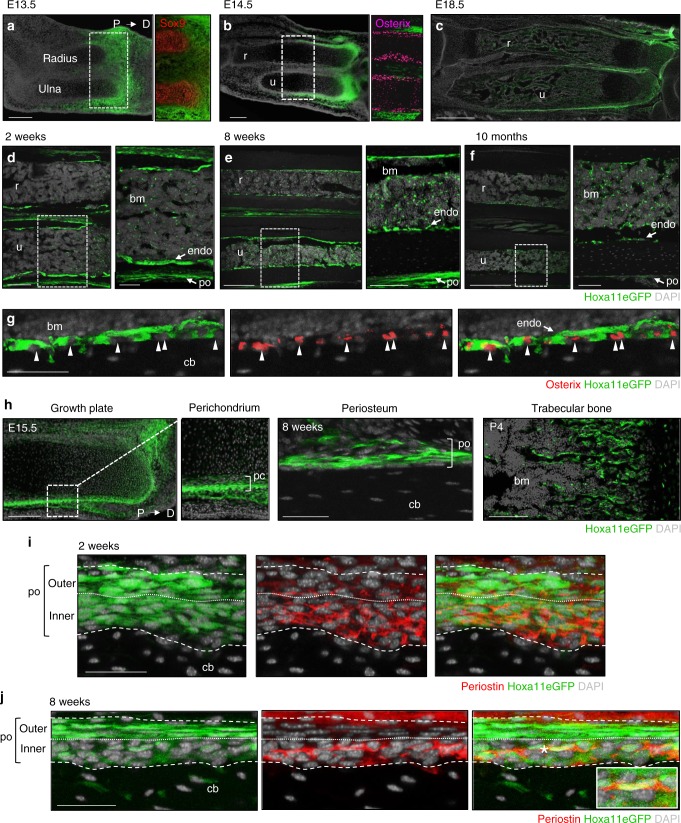
Hoxa11eGFP expression defines a continuous stromal population. **a**–**f** Hoxa11eGFP expression in the forelimb zeugopod (radius and ulna) shown from embryonic to adult stages with proximal on left and distal on right in all images. Hoxa11eGFP expression in radius and ulna **a**–**c**, higher magnification images show cartilage marker, Sox9 at E13.5 (**a**, red) and osteoblast marker, Osterix at E14.5 (**b**, magenta). **d**–**f** Mid-diaphysis radius (r) and ulna (u) (higher magnification images of mid-diaphysis ulna, white dashed box, shown on right). **g** Hoxa11eGFP and Osterix (red) at 8 weeks, white arrowheads identify individual Osterix positive nuclei. **h** Hoxa11eGFP (green) in E15.5 distal growth plate and higher magnification of perichondrium (white boxed region, bracket), 8 weeks periosteum (bracket), and P4 trabeculae. **i**, **j** Periosteal Hoxa11eGFP and Periostin (red) at 2 week (**i**) and 8 weeks (**j**). Dashed white lines mark periosteal boundary, dotted line separates inner and outer layers. **j** Cell marked by asterisk magnified in inset. In all images, green: Hoxa11eGFP, gray: DAPI. Bone marrow: bm, periosteum: po, endosteum: endo, cortical bone: cb, perichondrium: pc. Scale bars: (**a**, **b**, **d**–**f** left panels, **h** growth plate), 200 μm, (**c**) 500 μm, (**d**–**f** right panels, **h** trabecular bone) 100 μm, (**g**, **h** periosteum, **i**, **j**) 50 μm

*Hox11* expression is regionally restricted in the embryonic zeugopod limb (radius/ulna and tibia/fibula) and is observed in cells of the perichondrium surrounding the chondrocyte anlage (Fig. 1a). As osteoblast differentiation commences, *Hox11* continues to be expressed in the outer periosteum immediately adjacent to the differentiating osteoblast layer (Fig. 1b)^6^. Throughout embryonic, postnatal, and adult life, Hoxa11eGFP-expressing cells persist on the periosteal surface, but also are observed on the endosteal bone surfaces and as stromal cells within the bone marrow space beginning at postnatal stages (Fig. 1c–f). At later stages, Hoxa11eGFP-expressing cells remain non-overlapping with osteoprogenitors on the bone surfaces (Fig. 1g, arrowheads)^5^. We previously demonstrated that adult Hoxa11eGFP-expressing cells are exclusively identified by co-expression of PDGFRα/CD51 and of LepR, cell surface markers for progenitor-enriched MSCs^5,7,8^. Consistent with the possibility that Hox11 expression defines skeletal mesenchymal progenitors throughout life, Hoxa11eGFP-expressing cells are observed in several regions that have been demonstrated to contain skeletal progenitors including the distal growth plate, the perichondrium/periosteum, and the trabecular bone (Fig. 1h)^13,15–18^. Periostin expression was recently identified to mark MSCs with enriched bone-forming potential compared to bone marrow MSCs^19^. Intriguingly, Hoxa11eGFP-expressing cells in the outer periosteum are not positive for periostin at adolescent or adult stages, however, the more weakly postive Hoxa11eGFP cells in the inner periosteal layer do overlap with periostin staining, correlating the expression of both of these proteins with high progenitor activity in this region of the skeleton (Fig. 1i, j).

**Fig. 2:**
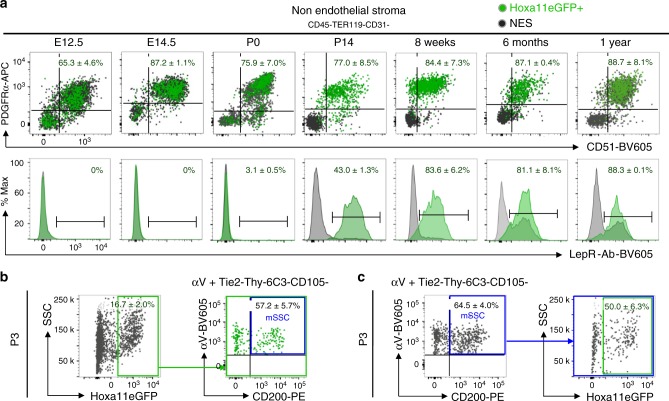
Hoxa11eGFP-expressing cells co-express MSC markers throughout life. **a** Flow cytometry analyses of whole skeletal anlage (E12.5 (*n* = 4), E14.5 (*n* = 6), and P0 (*n* = 6). ‘*n*’ values represent pooled cells from both forelimbs of individual embryos or pups) or flushed bone marrow (P14 (*n* = 9), 8 week (*n* = 10), 6 month (*n* = 4), and 1 year (*n* = 3). ‘*n*’ values represent pooled cells from the radius and ulna of one forelimb of individual animals). Gating strategy and bone surface analyses Supplementary Fig. 1a, b. Non-hematopoietic, non-endothelial stromal compartment (CD45-TER119-CD31-) was gated on PDGFRα/CD51 (top) or Leptin Receptor (LepR-Ab, bottom). Percentages reflect proportion of Hoxa11eGFP-positive population within double-positive gate (top) or bracketed region of histogram (bottom). Charcoal dots or gray histogram: total non-endothelial stroma (NES), green dots or green histogram: Hoxa11eGFP-expressing non-endothelial stroma (Hoxa11eGFP+). **b**, **c** Flow cytometry analyses of whole P3 bones [*n* = 3]. ‘*n*’ value represents pooled cells from the radius and ulna of both forelimbs of individual pups. Gating strategy Supplementary Fig. 1c. Non-hematopoietic, non-endothelial stromal compartment (CD45-TER119-CD31-) was gated for **b** Hoxa11eGFP-positive population (green box) and subsequently for mouse skeletal stem cells (mSSCs, αV+Tie2-Thy-6C3-CD105-CD200+, blue box) or **c** mSSC population (blue box) and subsequently for Hoxa11eGFP-expression (green box). Percentages reflect proportion of cells within indicated gate. ‘*n*’ values indicate biologically independent animals for each time point. All data presented as mean ± standard deviation

We analyzed Hoxa11eGFP-expressing cells from embryonic, postnatal, and adult stages for co-expression of PDGFRα/CD51 and LepR by flow cytometry. At embryonic stages, analyses were performed on the entire skeletal anlage, while at postnatal and adult stages, the bone marrow and bone adherent fractions were analyzed separately. Hoxa11eGFP-expressing cells from embryonic stages through 1 year of age co-label with PDGFRα and CD51 in both compartments (Fig. 2a and Supplementary Fig. 1b). In agreement with previous reports, LepR expression does not initiate until approximately newborn stages^8,10^. Consistent with increasing expression in stromal progenitors during postnatal life, co-expression of LepR in the Hoxa11eGFP-expressing cells increases during this time; by adult stages, the majority of Hoxa11eGFP-expressing cells are also LepR-positive (Fig. 2a and Supplementary Fig. 1b). Interestingly, LepR expression increases more slowly within the bone adherent compartment compared to the bone marrow compartment (compare Supplementary Fig. 1b to Fig. 2a). While it has not been established whether adult MSC cell-surface markers label progenitors during embryogenesis, Hoxa11eGFP-expressing stromal cells maintain a constant cell surface signature from the stage when each marker is first expressed. Primitive postnatal progenitors, coined mouse skeletal stem cells (mSSCs), are one of the earliest MSC populations defined by flow cytometry^20^. Overlap (50–60%) between Hoxa11eGFP-expressing cells and the mSSC population is observed demonstrating that a sub-population of postnatal Hox-expressing stromal cells are mSSCs (Fig. 2b, c). These collective data provide strong support for the hypothesis that Hoxa11eGFP-expression identifies a skeletal MSC population from early stages.

### Cas9/CRISPR generation of a *Hoxa11-CreER*^*T2*^ allele

To rigorously examine the lineage potential of Hox11-expressing cells in vivo, we generated a tamoxifen-inducible *Cre* knock-in at the *Hoxa11* locus using Cas9/CRISPR-mediated gene editing (Supplementary Fig. 2a). Briefly, two guide RNA sequences were designed to cut near the boundaries of exon 1 and a recombination plasmid was generated containing a tamoxifen-inducible *Cre* cassette (*CreER*^*T2*^) with the rabbit β-globin poly-adenylation sequence^21^. This recombinant template was flanked by 1.3 kb of homology upstream and downstream of exon 1. The editing strategy resulted in replacement of exon 1 with *CreER*^*T2*^ followed by a strong stop sequence while maintaining the endogenous *Hoxa11* surrounding sequences. Founder animals were screened by PCR for insertion of *Cre* sequence (Supplementary Fig. 2b). Targeting to the *Hoxa11* locus was validated by Southern Blot analyses using 5′ and 3′ flanking probes, as well as an internal probe for *Cre* (Supplementary Fig. 2c). *Hoxa11-CreER*^*T2*^ mice were crossed to *ROSA26-LSL-tdTomato* reporter mice and no tdTomato expression was observed in the absence of tamoxifen administration (Supplementary Fig. 2d).

### *Hoxa11*-lineage shows life-long contribution to skeleton

**Fig. 3:**
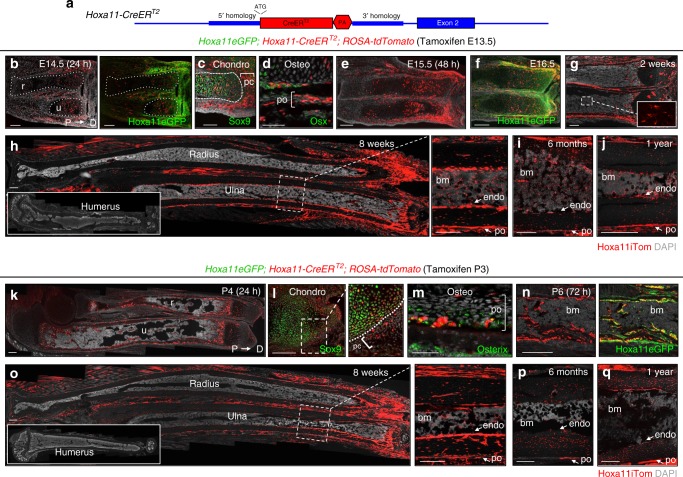
*Hoxa11*-lineage contributes to the zeugopod skeleton throughout life. **a** Schematic of *Hoxa11-CreER*^*T2*^ allele. Exon 1 of *Hoxa11* was replaced with *CreER*^*T2*^ followed by a rabbit β-globin poly-adenylation (PA) stop sequence (see “Methods” section). Endogenous sequence in blue, edited sequence in red, start site marked by ‘ATG’. **b**–**j** Pregnant females were given tamoxifen at E13.5 and resulting *Hoxa11eGFP;Hoxa11-CreER*^*T2*^*;ROSA-tdTomato* animals were examined at indicated ages. Shown are complete limb (**b**, **e**, **h**), distal radius (r) and ulna (u) (**f**, **g**) or distal diaphysis region of tibia (**i**, **j**). **c** Expression of Hoxa11iTom and chondrocyte marker, Sox9 (green) at E14.5, dashed white lines demarcates anlage and bracket marks perichondrium. **d** Expression of Hoxa11iTom and osteoblast marker, Osterix (green) at E14.5, bracket marks periosteum. **f** Co-expression of Hoxa11eGFP and Hoxa11iTom. **g** Inset shows *Hoxa11*-lineage marked bone marrow stromal cells (white dashed box). **k**–**q** P3 pups received tamoxifen and *Hoxa11eGFP;Hoxa11-CreER*^*T2*^;*ROSA-tdTomato* mice were examined at indicated ages. Shown are complete limb (**k**, **o**), mid-diaphysis ulna (**n**), or distal region of tibia (**p**, **q**). **l** Co-expression of Hoxa11iTom and chondrocyte marker, Sox9 (green) in the growth plate at P4 Boxed region enlarged to right, dashed white line demarcates perichondrial border. **m** Co-expression of Hoxa11iTom and osteoblast marker, Osterix (green) in the periosteum (bracket) at P4. **b**, **n** Right panel shows co-expression of Hoxa11iTom with Hoxa11eGFP. **h**, **o** Inset shows complete humerus. Dashed white box shown magnified to right; view of mid-diaphysis ulna. All images shown with distal end of bone to right. In all images, red: *Hoxa11*-lineage marked cells (Hoxa11iTom), green: Hoxa11eGFP-expressing cells (unless otherwise noted), gray: DAPI. Bone marrow: bm, perichondrium: pc, periosteum: po, endosteum: endo. Scale bars: (**c**, **n**) 100 μm, (**d**) 50 μm. All other scale bars = 200 μm

The in vivo lineage potential of Hox11-expressing cells was assessed by generating mice of the genotype *Hoxa11CreER*^*T2*^*;ROSA26-LSL-tdTomato* to report lineage contribution to the skeleton (Fig. 3a). A *Hoxa11eGFP* real-time reporter allele was also included in some animals to determine if *Hoxa11*-lineage marked cells persist as Hox11-expressing MSCs^22^. Lineage-tracing was initiated by administering tamoxifen to pregnant dams at E13.5, a time point at which embryonic Hoxa11eGFP expression has become restricted to the stromal population surrounding the condensed zeugopod cartilage, but several days prior to the formation of a bone marrow cavity (Fig. 3b)^6^. Embryonic *Hoxa11*-lineage-marked cells (*Hoxa11*^*E13.5*^) closely matched Hoxa11eGFP expression further confirming the integrity of the *Hoxa11-CreER*^*T2*^ allele (Fig. 3b). 24 h following tamoxifen injection, the majority of lineage-marked cells are localized within the perichondrial/periosteal stroma surrounding the skeletal element with little to no overlap with Sox9-postive chondrocytes and Osx-positive osteoblasts (Fig. 3c, d, brackets). 2 days after injection, at E15.5, the embryonic anlage has begun to mature, with cartilaginous growth plates on the distal ends and ossification initiating in the center of each skeletal element. *Hoxa11*^*E13.5*^-lineage-marked cells are observed throughout the perichondrium/periosteum surrounding the zeugopod elements and additionally within the growth plate and on the bone surface (Fig. 3e). Significant overlap of *Hoxa11*^*E13.5*^-lineage-marked cells and Hoxa11eGFP-expressing cells continues to be observed, while the *Hoxa11*-lineage-marked population has expanded (Fig. 3f and Supplementary Fig. 3a). By E18.5, *Hoxa11*^*E13.5*^-lineage-marked cells are observed throughout the zeugopod growth plate, within the primary spongiosa, and in the outer periosteal region (Supplementary Fig. 3b–d). *Hoxa11*^*E13.5*^-lineage-marked cells contribute to both growth plate chondrocytes and osteoblasts at E18.5, demonstrating that the Hox11-expressing population marks multipotent skeletal progenitors that function during embryogenesis (Supplementary Fig. 3b–d).

Following the *Hoxa11*^*E13.5*^-lineage-marked population after birth reveals continued lineage labeling throughout the periosteum, the endosteum, and within the established bone marrow space (Fig. 3g and inset). This pattern of distribution continues through adult stages where extensive lineage-labeling is observed (Fig. 3h). Consistent with the regional expression of Hoxa11eGFP, *Hoxa11*^*E13.5*^-lineage-marked cells only contribute to the zeugopod skeleton and no lineage labeling in the stylopod (humerus) is observed at any stage, demonstrating that this progenitor population remains regionally restricted (Fig. 3h, inset). *Hoxa11*^*E13.5*^-lineage-marked cells continue to show remarkably strong contribution to the skeleton as late as 1 year of age (Fig. 3i, j).

Lineage-labeling was then initiated at postnatal stages, a time when other genetic models have demonstrated contribution to long-lived stromal MSC cells. Tamoxifen was administered at postnatal day 3 (P3) and the contribution of *Hoxa11* lineage-marked cells (*Hoxa11*^*P3*^) was examined. During the first days following tamoxifen administration, *Hoxa11*^*P3*^-lineage-marked cells are observed within the perichondrium surrounding the distal growth plate and on the periosteal, the endosteal, and the trabecular bone surfaces (Fig. 3k). At 24 h following tamoxifen administration, *Hoxa11*^*P3*^-lineage-labeled cells are largely restricted to the periochondrium/periosteum and again show little overlap with Sox9-positive chondrocytes and Osx-positive osteoblasts (Fig. 3l, m, brackets). The pattern of *Hoxa11*^*P3*^-labeling shows clear overlap with Hoxa11eGFP expression (Fig. 3n). Following an 8-week chase, *Hoxa11*^*P3*^-lineage-marked cells contribute to the skeleton and are observed on periosteal and endosteal bone surfaces, as well as throughout the bone marrow space (Fig. 3o). Similar to E13.5 lineage-induction, *Hoxa11*^*P3*^-lineage-marked cells give rise to the skeletal lineages within the zeugopod and lineage contribution is not observed in the stylopod at this or any stage (Fig. 3o, inset). *Hoxa11*^*P3*^-lineage-marked cells persist and continue to contribute to the skeleton through 1 year of age (Fig. 3p, q). Of note, lineage induction at postnatal stages looks indistinguishable from embryonic induction, consistent with the Hox11-expressing population representing skeletal progenitors with equivalent capacity at both stages.

### *Hoxa11*-lineage becomes all skeletal/mesenchymal cell types

**Fig. 4:**
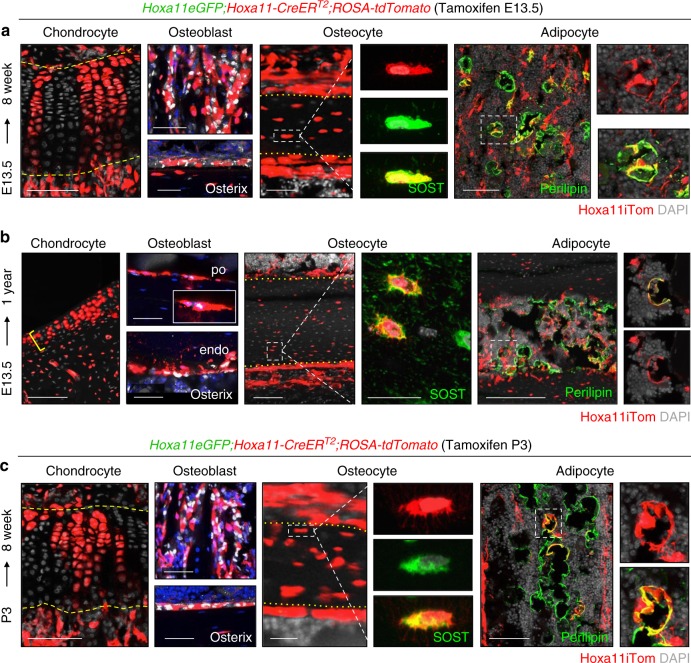
*Hoxa11-*lineage contributes to all skeletal/mesenchymal cell types. **a**–**b** Pregnant dams received tamoxifen at E13.5 and resulting *Hoxa11eGFP;Hoxa11-CreER*^*T2*^*;ROSA-tdTomato* mice were chased to (**a**) 8 weeks or (**b**) 1 year. **c** P3 pups received tamoxifen and *Hoxa11eGFP;Hoxa11-CreER*^*T2*^*;ROSA-tdTomato* mice were chased to 8 weeks. All images, *Hoxa11* lineage-labeled cells (Hoxa11iTom, red). Shown are chondrocytes with characteristic columnar morphology at distal end of growth plate, osteoblasts stained with Osterix (white) on trabecular (top) and endosteal bone (bottom), osteocytes within the cortical bone stained with SOST (green), and bone marrow adipocytes stained with Perilipin (green). Dashed yellow lines mark upper and lower boundaries of growth plate, dotted yellow lines mark periosteal and endosteal boundaries of cortical bone. White dashed boxed region of single (**a**, **c**) or multiple (**b**) osteocytes(s) magnified to right. White dotted box of single adipocyte magnified to right. In all images, gray or blue: DAPI. Scale bars: (chondrocyte and adipocytes images) 100 μm, (osteoblast and osteocyte images) 50 μm, (**b**, SOST) 25 μm

To assess the contribution of *Hoxa11-CreER*^*T2*^ lineage-marked cells to differentiated mesenchymal skeletal cell types at adult stages, we performed co-labeling with markers for cartilage, bone, and adipose tissues. *Hoxa11*^*E13.5*^-lineage-marked cells can be visually identified as differentiating into chondrocytes within the growth plate, co-staining with markers of differentiation identifies *Hoxa11*^*E13.5*^-lineage cells as osteoblasts on the trabecular and endosteal bone surfaces, osteocytes embedded within the cortical bone, and as adipocytes in the bone marrow (Fig. 4a). Analysis out to 1 year of age shows that *Hoxa11*^*E13.5*^-lineage-marked cells continue to give rise to all skeletal lineages; chondrocytes, osteoblasts, osteocytes, as well as bone marrow adipocytes (Fig. 4b). At these stages, the growth plate has collapsed in mice, however *Hoxa11*^*E13.5*^-lineage-marked chondrocytes are observed throughout the articular cartilage (Fig. 4b, yellow bracket). It is important to note that, in adult mice, osteoblasts are reported to live for one month, therefore multiple rounds of osteoblast turnover have presumably occurred between E13.5 and 1 year of age^23–26^.

The same analyses were performed on *Hoxa11*^*P3*^-lineage-marked stromal cells where essentially identical results are observed. *Hoxa11*^*P3*^-lineage-marked cells differentiate into all mesenchymal skeletal cell types, including chondrocytes, osteoblasts, osteocytes, and bone marrow adipocytes (Fig. 4c). *Hoxa11*^*P3*^-lineage-marked cells also continue to mark the same populations through 1 year of age (Supplementary Fig. 4a).

### *Hoxa11*-marked progenitors are maintained throughout life

**Fig. 5:**
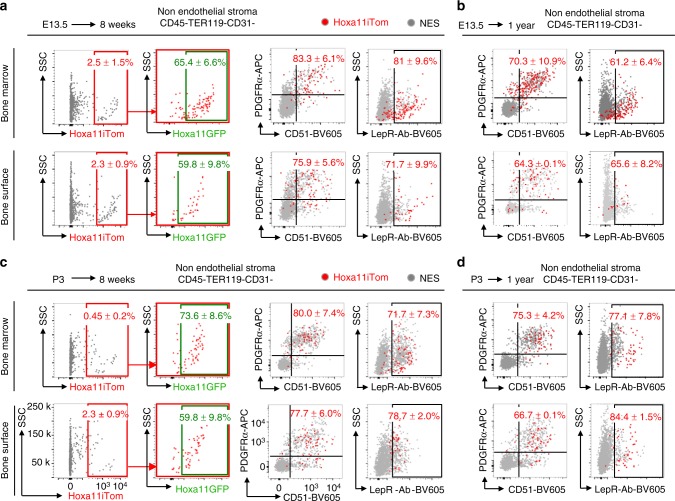
*Hoxa11*-lineage-marked progenitors are maintained throughout life. (**a**, **b**) Pregnant dams received tamoxifen at E13.5 and resulting *Hoxa11eGFP;Hoxa11-CreER*^*T2*^*;ROSA-tdTomato* mice were chased to (**a**) 8 weeks (*n* = 3) or (**b**) 1 year (*n* = 4). P3 pups received tamoxifen and *Hoxa11eGFP;Hoxa11-CreER*^*T2*^*;ROSA-tdTomato* mice were chased to (**c**) 8 weeks (*n* = 6) or (**d**) 1 year (*n* = 3). Flow cytometry analyses of non-hematopoietic, non-endothelial stromal compartment (CD45-TER119-CD31-, NES) in bone marrow (top panels), and bone surface (bottom panels). **a**, **c** First panel: *Hoxa11* lineage-marked cells (*x*-axis: Hoxa11iTom), second panel: analysis of Hoxa11iTom positive gate (red) for Hoxa11eGFP expression, third panel: co-expression analysis of PDGFRα/CD51, fourth panel: co-expression analysis of Leptin Receptor (LepR-Ab). **b**, **d** Left: co-expression analysis of PDGFRα/CD51, right: co-expression analysis of Leptin Receptor (LepR-Ab). Percentages reflect proportion of Hoxa11iTom population in identified gate. Gray dots: total non-endothelial stroma (NES), red dots: Hoxa11iTom. ‘*n*’ values indicate pooled cells from radius and ulna of one forelimb from biologically independent animals for each time point. All data presented as mean ± standard deviation

We next performed flow cytometry analyses on *Hoxa11*^*E13.5*^-lineage-marked stromal cells from the zeugopod bone marrow and bone surfaces to assess the cell surface identity of these cells. The majority of *Hoxa11*^*E13.5*^-lineage-marked stromal cells from both compartments were confined to the non-hematopoietic, non-endothelial stromal compartment (CD45-TER119-CD31-) (Supplementary Fig. 3e). Adult, *Hoxa11*^*E13.5*^-lineage-marked bone marrow stromal cells specifically express MSC markers PDGFRα/CD51 and LepR, demonstrating that the embryonically labeled Hoxa11-expressing cells give rise to adult, progenitor-enriched MSCs that are maintained throughout life (Fig. 5a). Similar flow cytometry profiles are observed for *Hoxa11*^*E13.5*^-lineage-marked cells that persist on the cortical bone surfaces, with the majority of labeled bone surface stromal cells co-expressing PDGFRα/CD51 and LepR as we have previously reported for real-time expression of Hoxa11eGFP (Fig. 5a)^5^. *Hoxa11*^*E13.5*^-lineage-marked cells continue to co-express these MSC markers through 1 year of age and beyond (Fig. 5b). Additionally, a majority of *Hoxa11*^*E13.5*^-lineage-marked cells co-express Hoxa11eGFP (Fig. 5a and Supplementary Fig. 3f, g). Collectively these data provide evidence that the embryonic Hoxa11-expressing cell population gives rise to Hoxa11eGFP-expressing MSCs that persist throughout the life of the animal.

The same analyses were performed on *Hoxa11*^*P3*^-lineage-marked stromal cells and nearly identical results are observed. Flow cytometry analyses on adult, *Hoxa11*^*P3*^-lineage-marked bone marrow and bone surface stromal cells demonstrate that *Hoxa11*^*P3*^-lineage-marked cells are contained within the non-hematopoietic, non-endothelial stromal compartment, and co-express progenitor-enriched MSC markers PDGFRα/CD51 and LepR in both the bone marrow and bone surface compartments and continue to co-express these MSC markers out to 1 year of age (Fig. 5c, d and Supplementary Fig. 4b). *Hoxa11*^*P3*^-lineage-marked cells also express Hoxa11eGFP in both compartments (Supplementary Fig. 4c).

### *Hoxa11*-lineage regenerates bone and cartilage upon fracture

**Fig. 6:**
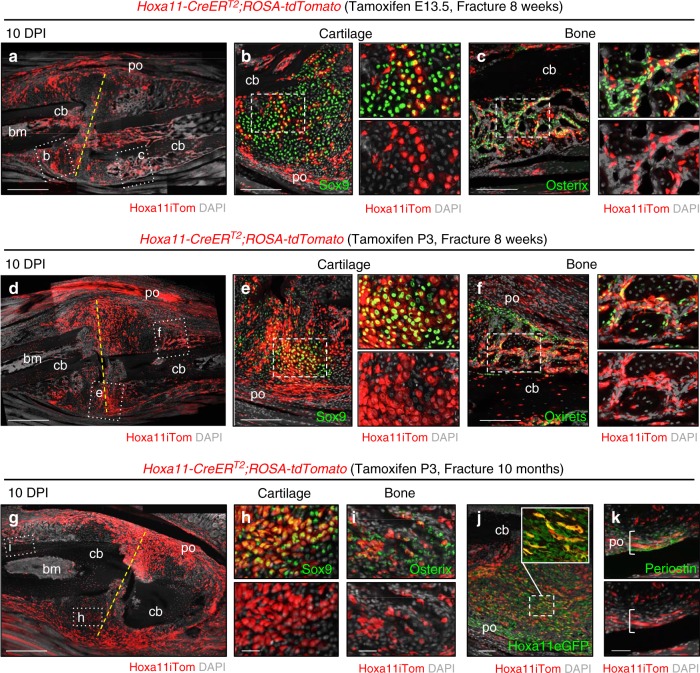
*Hoxa11*-lineage cells regenerate skeleton following fracture. Ulnar fracture was performed at 8 weeks of age (**a**–**f**) or 10 months (**g**–**k**) on *Hoxa11-CreER*^*T2*^*;ROSA-tdTomato* or *Hoxa11eGFP;Hoxa11-CreER*^*T2*^*;ROSA-tdTomato* mice following tamoxifen dosing at (**a**–**c**) E13.5—to pregnant female, or (**d**–**k**) P3. **a**, **d**, **g** *Hoxa11* lineage-positive cells (Hoxa11iTom, red) within the fracture callus 10 days post-injury (DPI). Fracture line marked with dashed yellow line and cortical bone (cb), bone marrow (bm), and periosteum (po) are labeled. Dashed white lines indicate regions visualized with antibodies (green) for cartilage (Sox9, **b**, **e**, **h**) and bone (Osx, **c**, **f**, **i**). Higher magnification of regions marked by dashed white lines (**b**, **c**, **e**, **f**) with channels separated shown to right. (**j**) Expression of Hoxa11eGFP (green) and Hoxa11iTom within the expanded stromal population. White dashed box magnified in inset. **k** Expression of Periostin (green) and Hoxa11iTom within the expanded periosteal compartment (bracket). In all images, gray: DAPI. Scale bars: (**a**, **d**, **g**) 500 μm, (**b**, **c**, **e**, **f**) 200 μm, (**j**) 100 μm, (**h**, **i**, **k**) 50 μm

To examine whether *Hoxa11-*lineage-marked cells serve as progenitors in regeneration and repair of the skeleton following injury, *Hoxa11CreER*^*T2*^*;ROSA26-LSL-tdTomato* animals were administered tamoxifen at E13.5 or at P3 to initiate lineage-labeling and the ulna was fractured at adult stages (8–10 weeks of age). Contribution to regenerating cartilage and bone was analyzed 10 days post-injury (10 DPI). *Hox11*^*E13.5*^- and *Hoxa11*^*P3*^-lineage marked cells expand in response to fracture and are observed throughout the callus (Fig. 6a, d). Apparent expansion of both the periosteal stromal compartment and the bone marrow stromal compartment is observed. *Hoxa11*-lineage-marked cells give rise to both Sox9-positive chondrocytes within the cartilaginous regions of the callus and to Osx-expressing osteoblasts within the woven bone regions of the callus (Fig. 6b, c, e, f). Contribution of *Hoxa11*-lineage-marked cells to the regenerating skeletal tissues is qualitatively equivalent whether lineage-tracing is initiated at embryonic or postnatal stages, demonstrating that Hoxa11-positive cells from both stages represent roughly equivalent, functional, adult skeletal MSCs.

To test the continued functionality of the *Hoxa11*-lineage-marked progenitor population throughout life, we allowed lineage-marked animals to age to 10 months prior to ulnar fracture. At late adult stages, the *Hoxa11*^*P3*^-lineage-marked cell population expands robustly following injury and contributes to regenerating cartilage and bone 10DPI (Fig. 6g–i). Additionally, the expanded lineage-marked stromal cells are Hoxa11eGFP-positive, representing expanding progenitors as shown previously (Fig. 6j)^5^. Lineage-marked cells within the periosteal region of the fracture callus are also periostin-positive, consistent with expansion of Hox11-expressing periosteal stem cells following injury (Fig. 6k).

### Comparison of *Hoxa11*-lineage to other MSC populations

A critical knowledge gap in the field is an understanding of the relationships between various identified MSC progenitor populations. Significant differences exist in the lineage-dynamics of these populations, yet the reasons for these differences are not clearly understood. We sought to establish the relationship between Hox11-expressing cells and previously reported MSC populations genetically labeled by *LepR-Cre* and *Osx-CreER* using our *Hoxa11eGFP* real-time reporter crossed to *LepR-Cre;ROSA26-LSL-tdTomato* mice or *Osx-CreER;ROSA26-LSL-tdTomato* mice.

**Fig. 7:**
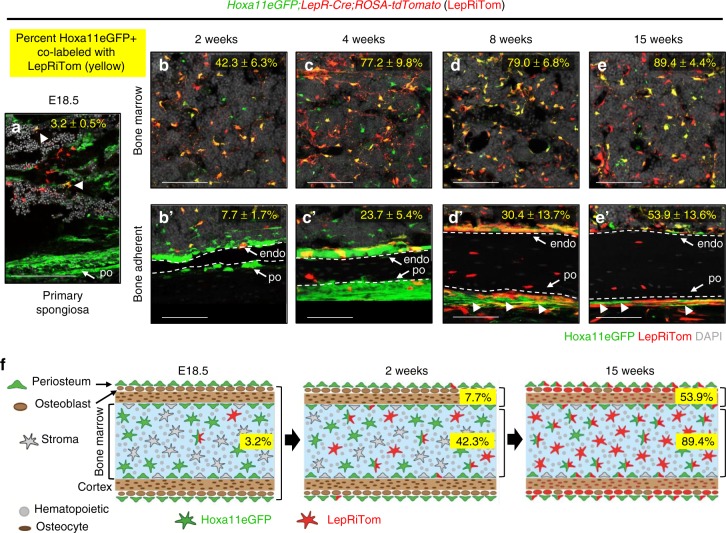
*LepR-Cre* progressively marks existing Hoxa11eGFP-positive cells. Analysis of the co-expression of Hoxa11eGFP (green) and LepR-Cre lineage-marked cells (LepRiTom, red) in *Hoxa11eGFP;LepR-Cre;ROSA-tdTomato* mice. Percentages (yellow) reflect proportion of Hoxa11eGFP-positive cells that also express LepRiTom. Flow cytometry analyses shown in Supplementary Fig. 5. **a** magnified view of primary spongiosa and developing cortical bone surface at E18.5 (*n* = 3). **b**–**e′** High magnification view of mid-diaphysis of ulna **b**–**e** bone marrow, or **b′**–**e′** cortical bone at 2 (*n* = 4), 4 (*n* = 3), 8 (*n* = 3), and 15 weeks (n = 3). **d**′, **e**′ Arrowheads identify non-overlapping Hoxa11eGFP-positive periosteal cells. In all images; gray: DAPI. Data presented as mean ± standard deviation. ‘*n*’ values indicate pooled cells from **a** both E18.5 forelimbs or **b**–**e′** the radius and ulna of one forelimb of biologically independent animals for each time point. **f** Diagram of data at E18.5, 2 weeks, and 15 weeks. All scale bars: 100 μm

*LepR-Cre* lineage-marked cells first appear during late embryonic stages within the primary spongiosa (Fig. 7a)^8,10^. At this stage, Hoxa11eGFP-expressing cells are more extensively observed throughout the periosteum and along the bone surfaces of the primary spongiosa. There is only very rare overlap between *LepR-Cre* lineage-marked cells and Hoxa11eGFP at early stages (Fig. 7a, arrowheads). The number of *LepR-Cre* lineage-marked cells increases markedly during postnatal development. In the bone marrow, the overlap between Hoxa11eGFP and *LepR* lineage-marked cells progressively increases with age with more of the Hoxa11eGFP-positive cells becoming LepR-positive (Fig. 7b–e and Supplementary Fig. 5). By 15 weeks, the majority of Hoxa11eGFP-expressing bone marrow stromal cells are also *LepR*-lineage positive (Fig. 7e and Supplementary Fig. 5)^5^. The number of *LepR-Cre* lineage-marked cells on the periosteal and endosteal bone surfaces also increases with age, however even as late as 15 weeks of age, only half of Hoxa11eGFP, bone-adherent cells are *LepR*-lineage positive (Fig. 7b′–e′ and Supplementary Fig. 5). Interestingly, there is a population of Hoxa11eGFP-expressing cells on the outer perichondrial surface that remains *LepR-Cre*-lineage-negative at all stages examined (Fig. 7d′, e′, arrowheads). These results are consistent with *LepR*-lineage labeling continuing to initiate in Hoxa11-positive cells (Fig. 7f). Differences in the overlap between the bone marrow and bone-adherent populations reveal a unique Hoxa11-expressing population in the periosteum, perhaps explaining, at least in part, the earlier and more extensive *Hoxa11*-lineage contribution to differentiated skeletal cells.

**Fig. 8:**
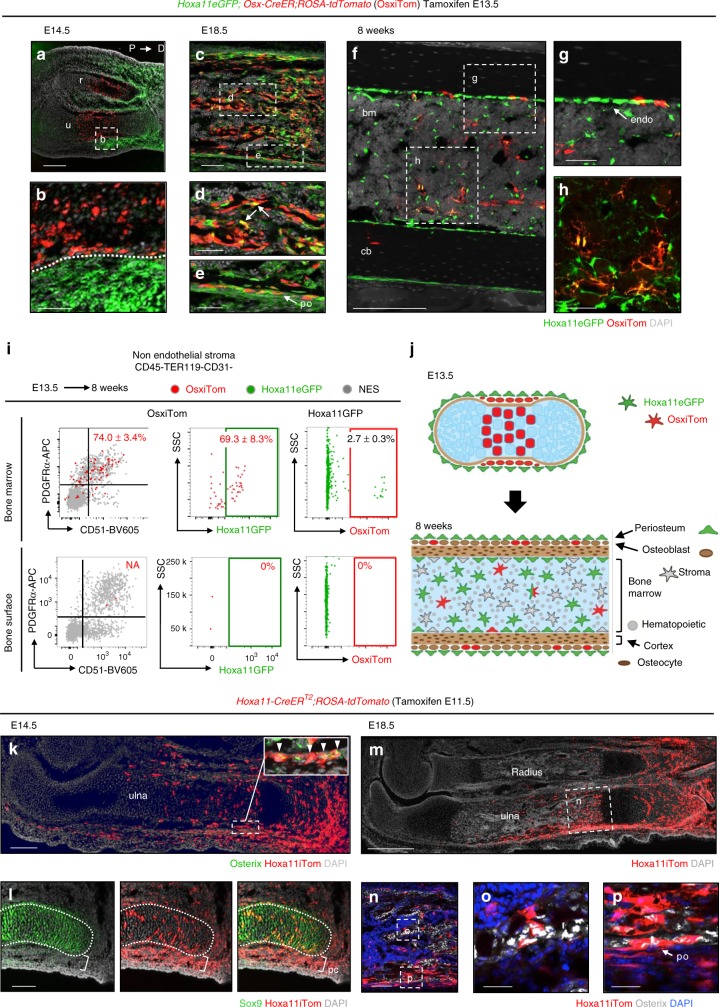
Hoxa11eGFP-positive MSCs are distinct from embryonic *Osx-*lineage. Comparison of Hoxa11eGFP (green) and *Osx-CreER* lineage-marked cells (OsxiTom, red) was performed in *Hoxa11eGFP;Osx-CreER;ROSA-tdTomato* mice. **a**–**i** Pregnant females received tamoxifen at E13.5 and co-expression of Hoxa11eGFP and embryonic *Osx*-lineage was examined at (**a**, **b**) E14.5, (**c**, **d**) E18.5, and (**f**–**i**) 8 weeks. **a** Complete radius (r) and ulna (u) 24 h after tamoxifen with proximal to the left and distal to the right. White box region magnified in (**b**) anlagen boundary indicated by dotted white line. **c** Mid-diaphysis ulna; white dashed boxes indicate magnified region of (**d**) primary spongiosa and (**e**) periosteum. **f** Mid-diaphysis tibia; white dashed boxes indicate magnified region of adult (**g**) endosteal surface and (**h**) bone marrow. (**i**) Flow cytometry analysis of non-hematopoietic, non-endothelial stroma (CD45-TER119-CD31-, NES) in bone marrow (top panels) and bone adherent (bottom panels) compartments. First panel: analysis of OsxiTom population for PDGFRα/CD51, second panel: analysis of OsxiTom for Hoxa11eGFP, third panel: analysis of Hoxa11eGFP-positive cells (green) for OsxiTom expression. OsxiTom cells are very rare (0.018 ± 0.03 of total NES) on bone surface (lower left panel). (*n* = 3). ‘*n*’ represents pooled cells from the radius and ulna of one forelimb of biologically independent animals. Flow cytometry dot plots, gray dots: total non-endothelial stroma (NES). Data presented as mean ± standard deviation. **j** Diagrammatic representations of data. **k** Pregnant females received tamoxifen at E11.5 and *Hoxa11-CreER*^*T2*^*;ROSA-tdTomato* embryos were analyzed at (**k**, **l**) E14.5 and (**m**–**p**) E18.5. **k** White boxed region shown in inset, co-expression of Hoxa11iTom (red) and Osterix (green). (**m**) white boxed region of primary spongiosa magnified in **n**. **n** White dashed boxes indicate magnified regions of (**o**) trabeculae and (**p**) periosteum. Periosteum: po, bone marrow: bm, cortical bone: cb, endosteum: endo All fluorescent images, gray: DAPI. Scale bars: (**a**, **f**, **k**) 200 μm, (**b**, **d**–**e**, **g**–**h**) 50 μm, (**c**, **i**, **n**) 100 μm, (**o**, **p**) 25 μm

Previous reports demonstrate O*sx*-lineage contribution to MSCs when induction is initiated at postnatal stages but the absence of long-term lineage contribution when induced at embryonic stages^10,11^. We induced *Osx*-lineage labeling in *Hoxa11eGFP;Osx-CreER;ROSA26-LSL-tdTomato* animals at E13.5 or at P3 to compare these populations to Hoxa11eGFP-expressing cells. 1 day following tamoxifen administration at E13.5, embryonic *Osx*-lineage (*Osx*^*E13.5*^)-marked cells are restricted to the inner periosteal, pre-osteoblast layer, while Hox11-expressing cells are restricted to the outer periosteal layer with little to no overlap observed (Fig. 8a, b). *Osx*-*CreER* lineage induction at E13.5 also labels hypertrophic chondrocytes in the developing bone. The pattern of *Osx*^*E13.5*^*-*lineage marked cells is consistent with Osterix protein expression using an anti-Osterix antibody (compare Fig. 8b and Fig. 1b). By E18.5, *Osx*^*E13.5*^*-*lineage-marked cells are observed throughout the primary spongiosa and minimal co-expression with Hoxa11eGFP is observed (Fig. 8c–e, arrows). Hoxa11eGFP-expressing cells and *Osx*^*E13.5*^*-*lineage-marked cells continue to exhibit an almost mutually exclusive stratified pattern in the periosteum, with *Osx*^*E13.5*^*-*lineage-marked cells in the inner pre-osteoblast layer and Hoxa11eGFP the adjacent outer periosteal layer (Fig. 8e). *Osx*^*E13.5*^-lineage-marked cells and Hoxa11eGFP-expressing cells show a small degree of overlap in the adult bone marrow and on the endosteal bone surface (Fig. 8f–i). However, *Osx*^*E13.5*^*-*lineage-marked cells are rare at adult time points and represent only 2.7 ± 0.3% of the total Hoxa11eGFP-positive population (Fig. 8i). Flow cytometry analyses of the small population of bone marrow *Osx*^*E13.5*^*-*lineage-marked cells shows 74.0 ± 3.4% co-express PDGFRα/CD51 and 69.3 ± 8.3% co-expresses Hoxa11eGFP (Fig. 8i). Virtually no *Osx*^*E13.5*^*-*lineage-marked cells remain on the bone surfaces by adult stages, having presumably differentiated into osteoblasts and osteocytes by 8 weeks (Fig. 8g, i). As these two populations do not progressively overlap with age, these data reveal that the Hoxa11eGFP-positive perichondrial/periosteal population is distinct from the embryonic *Osx*^*E13.5*^*-*lineage-marked cells (Fig. 8j).

At the earliest stages of limb development, Hox11 expression is not confined to the zeugopod but is broadly expressed in the lateral plate mesoderm^6^. Tamoxifen was administered to *Hoxa11-CreER*^*T2*^*;ROSA-tdTomato* animals at E11.5 (*Hox11*^*E11.5*^), 2 days prior to initiation of Osterix expression in the developing skeleton, and the contribution of *Hoxa11*^*E11.5*^-lineage-marked cells to the skeleton was assessed. At E14.5, *Hoxa11*^*E11*.5^-lineage marked cells have contributed significantly to the developing skeleton including Osx-positive osteoprogenitors within the developing periosteum (Fig. 8k, inset). There is additionally contribution to Sox9-positive chondro-progenitors throughout the distal growth plate consistent with Hox11-expressing cells serving as upstream progenitors for this population as well, as expected for a broad marker of lateral plate mesoderm (Fig. 8l). *Hoxa11*^*E11.5*^-lineage-marked cells continue to contribute extensively to the developing skeleton, including significant contribution to osteoblasts on the trabecular and cortical bone surfaces (Fig. 8m–p). These data show that the *Hoxa11*-lineage arises prior to the *Osx*-lineage, and that Hox11-expressing cells serve as primitive progenitors that give rise to early osteoprogenitor and the osteoblast lineage.

**Fig. 9:**
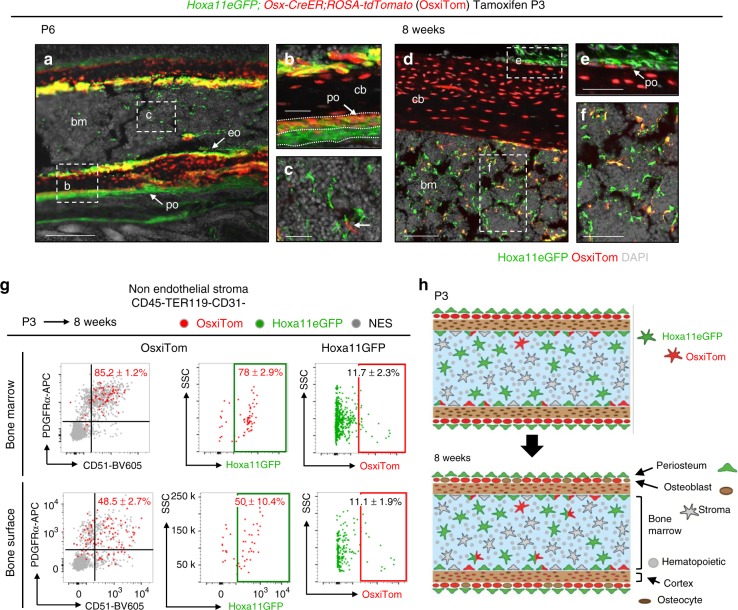
Postnatal *Osx-*lineage marginally overlaps with Hoxa11eGFP-positive cells. Comparison of Hoxa11eGFP (green) and *Osx-CreER* lineage-marked cells (OsxiTom, red) was performed in *Hoxa11eGFP;Osx-CreER;ROSA-tdTomato* mice. P3 pups received tamoxifen and co-expression of Hoxa11eGFP and postnatal *Osx*-lineage was examined at (**a**–**c**) P6 and (**d**–**f**) 8 weeks. (**a**) Mid-diaphysis ulna; dashed white boxes indicate magnified (**b**) cortical bone and (**c**) bone marrow, rare OsxiTom stromal cell (arrow). (**d**) Mid-diaphysis tibia, white dashed boxes indicate magnified (**e**) periosteum and (**f**) bone marrow. (**g**) Flow cytometry analysis of non-hematopoietic, non-endothelial stroma (CD45-TER119-CD31-) in bone marrow (top panels) and bone surface (bottom panels) compartments. First panel: analysis of OsxiTom population for PDGFRα/CD51, second panel: analysis of OsxiTom for Hoxa11eGFP, third panel: analysis of Hoxa11eGFP-positive cells (green) for OsxiTom expression. (*n* = 4). ‘*n*’ represents pooled cells from the radius and ulna of one forelimb of biologically independent animals. Flow cytometry dot plots, gray dots: total non-endothelial stroma (NES). Data presented as mean ± standard deviation. (**h**) Diagrammatic representations of data. Periosteum: po, bone marrow: bm, cortical bone: cb, endosteum: endo All fluorescent images, gray: DAPI. Scale bars: (**a**) 200 μm, (**b**, **c**, **e**, **f**) 50 μm, (**d**) 100 μm

Consistent with the reported progenitor capacity of the postnatal *Osx*-lineage-marked population (*Osx*^*P3*^), some overlap is observed between Hoxa11eGFP-expressing cells and *Osx*^*P3*^*-*lineage-marked cells at this stage. 3 days following tamoxifen administration, *Osx*^*P3*^*-*lineage-marked cells are localized to the bone surfaces (periosteal, endosteal, and trabecular) (Fig. 9a, b). The Hoxa11eGFP-expressing cells that are observed in the periosteum overlap with *Osx*^*P3*^*-*lineage-marked cells at the innermost periosteal layer, but additional non-overlapping Hoxa11eGFP-positive cells are observed in the outer periosteum (Fig. 9b). Hoxa11eGFP-positive cells are also already present at this stage throughout the bone marrow space and no co-expression with rare *Osx*^*P3*^*-*lineage-marked bone marrow cells is observed (Fig. 9c, arrow). Following an 8-week chase, *Osx*^*P3*^*-*lineage-marked cells contribute to bone, fat, and bone marrow stromal cells, consistent with previous reports (Fig. 9d–f)^10^. Most *Osx*^*P3*^*-*lineage-marked cells in the bone marrow co-express Hoxa11eGFP and markers for progenitor-enriched MSCs, PDGFRα/CD51 (Fig. 9f, g). On the bone surfaces, only about half of *Osx*^*P3*^*-*lineage-marked cells co-express PDGFRα/CD51 and Hoxa11eGFP (Fig. 9e, g). The remaining population likely reflects lineage-marked pre-osteoblasts. Interestingly, the majority of overlap between Hoxa11eGFP-expressing cells and *Osx*^*P3*^*-*lineage-marked cells is observed on the endosteal surface, while a stratified, non-overlapping expression pattern continues to be observed on the periosteal surface (Fig. 9d, e). Of note, *Osx*^*P3*^*-*lineage-marked cells at adult stages represent only a small fraction of the total Hoxa11eGFP-positive population, ~11% (Fig. 9g). These data show that *Osx-CreER* marks a sub-population of Hox11-expressing MSCs at postnatal stages that persist to adult stages (Fig. 9h). These data additionally highlight the unique Hox11-expressing population in the outer periosteum, adjacent to the Osx-positive inner pre-osteoblast periosteal layer that does not overlap with *Osx*-lineage-marked cells at any stage examined.

## Discussion

Previous lineage analyses support a model whereby transient, embryonic, skeletal progenitors are replaced with bona fide adult skeletal stem cells that are established during early postnatal life^27,28^. A substantial caveat to many of these genetic tools is that tamoxifen-inducible *Cre* expression is driven by promoters for genes that function in early lineage commitment to skeletal cell types (for instance chondrocyte—*Sox9-CreER*, *PthrP-CreER*, and *Aggrecan-CreER*, or osteoblast—*Osx-CreER*),^10,11^ (reviewed by Mendez-Ferrer et al. ^29^). Therefore, the bulk of these lineage-marked cells will be committed to the chondrocyte or osteoblast fate while only a small population of progenitors will be marked by these alleles. Labeling of long-term skeletal progenitors by these models is incomplete and the temporal differences in MSC capacity observed likely reflects depletion of early committed progenitors over time. Other genetic models, driven by promoters of genes involved in key signaling pathways, such as Hedgehog (*Gli1-CreER*), BMP (*Gremlin1-CreER*), or Parathyroid hormone-related protein (*PthrP-CreER*), also demonstrate temporal differences in progenitor capacity over time^12–14^. These signaling pathways are important for chondrogenic and osteogenic differentiation and differences in the progenitor capacity of these lineage-marked populations with time may reflect changes in the signaling environment of the skeleton and reporter/lineage expression, in these cases, would not be expected to specifically label the skeletal progenitor pool^30–33^. Collectively, the caveats to these models suggest that the proposed waves of skeletal progenitors may be an artifact of the genetic models and not a physiologically relevant property of skeletal progenitors.

Prior reports establish that *Hox* expression is excluded from differentiated skeletal cell types at all stages and loss-of-function analyses at embryonic, postnatal, and adult stages provide evidence for *Hox* gene function in the skeleton throughout life^3–6^. Herein, we present evidence that Hox11-expressing stromal cells, marked by *Hoxa11-CreER*^*T2*^ from both embryonic and postnatal stages, specifically enrich for a population of skeletal progenitors throughout the life of the animal and are likely to encompass a bona fide skeletal stem cell population. In contrast to models asserting that developmental progenitors are later replaced by postnatally arising adult MSCs, these data reveal a lineage-continuous population that is maintained from embryonic through adult stages. *Hoxa11* lineage-marked cells contribute extensively to the skeleton, giving rise to all skeletal cell types including chondrocytes, osteoblasts, osteocytes, and bone marrow adipocytes at all stages examined. *Hoxa11* lineage-marked cells additionally persist within the bone marrow space and on the cortical bone surfaces throughout life and maintain co-expression of MSC markers. Further, Hoxa11eGFP/*Hoxa11* lineage-marked cells expand following fracture injury and contribute to regenerating cartilage and bone, even when injury is induced at late adult stages, demonstrating that Hox11-expressing MSCs functionally give rise to new skeletal cells throughout life. The collective evidence demonstrates a continuous lineage relationship between the embryonic and the adult Hox11-expressing skeletal progenitor, MSC population. The data provides in vivo evidence that the Hox11-expressing stromal population enriches for life-long, self-renewing skeletal MSCs.

Assessing the relationship between different genetically defined populations of skeletal progenitors is critical for understanding how these populations behave and can provide information on the spatiotemporal dynamics of skeletal stem cells. *Hoxa11*-lineage-marked progenitors arise days prior to the differentiation of pre-osteoblasts (as defined by *Osx* expression that begins ~E13.5) or *LepR-Cre* (initiates ~E17.5). Given *LepR-Cre* is not a temporally controlled Cre, the progressive overlap of Hoxa11eGFP and *LepR*-lineage-marked cells suggest that *LepR-Cre* transgenic expression, and thus, the *LepR*-lineage, is increasingly initiated within the Hoxa11eGFP-positive population. Also, considering the adjacent but non-overlapping organization of Hoxa11eGFP-expressing cells and *Osx*-lineage-marked cells in the periosteum, these data lead to the conclusion that Hox-expressing progenitors in the outer periosteum give rise to the population marked by *Osx-CreER*, and subsequently the complete osteo-lineage. These comparative experiments demonstrate that the *Hoxa11eGFP*-expressing stromal population serves as the upstream population that gives rise to populations labeled by *Osx-CreER* and *LepR-Cre*.

Examination of the spatial overlap between Hoxa11eGFP-expressing cells and the populations marked by *Osx-CreER* or *LepR-Cre* revealed a Hoxa11eGFP-expressing population that is uniquely present in the outer periosteum. This population is non-overlapping with either the *LepR* lineage-marked population or the *Osx-CreER*-marked population after short-term or long-term lineage-labeling. The periosteal compartment has recently been shown to contain skeletal stem cells, identified by expression of periostin, with greater capacity to regenerate bone compared to bone marrow MSCs^19^. Hox11-expressing cells in the inner periosteal layer overlap with the pattern of periostin expression, at homeostasis and following fracture, providing evidence to corroborate that this spatially defined sub-population of periosteal cells may be, at least in part, the bona fide skeleton stem cell population. Through a series of sophisticated transplantation studies, Duchamp de Lageneste et al. demonstrated that adult periosteal and adult bone marrow MSCs both derive from the local embryonic perichondrial/periosteal mesenchyme^19^. Our *Hoxa11*-lineage tracing data provides direct in vivo evidence that is in complete concordance with this conclusion.

We previously reported that adult bone marrow MSCs from different anatomical regions display a differential Hox expression profile. Specifically, each skeletal compartment maintains the *Hox* expression that is established during embryonic development^5^. Importantly, *Hox* expression in the adult bone marrow is confined to only progenitor-enriched MSCs. *Hoxa11-CreER*^*T2*^ allows for the unique, in vivo labeling of the zeugopod-restricted MSC population and shows that skeletal contributions of MSCs remain regional throughout life. Skeletal MSCs are not mobile; they remain at or near their site of origin. Our data support a model whereby regional, Hox-expressing stem cell populations in the skeleton are established during embryonic development and give rise to regionally restricted, skeletal mesenchymal stem cells that self-renew and function throughout the life of the animal.

## Methods

### Mouse models

All mice were maintained in a C57BL/6 background. *Hoxa11eGFP*^22^, *LepR-Cre*^34^, *Osterix-CreER*^*T2*^ ^16^ mice have been described elsewhere. Rosa26-CAG-loxp-stop-loxp-tdTomato^35^ (JAX stock #007909) were obtained from Jackson Laboratory. Both male and female mice were used for experiments. Animals were sacrificed for experiments through CO_2_ exposure followed by removal of a vital organ. All procedures described here were conducted in compliance with the University of Michigan’s Committee on Use and Care of Animals, protocol PRO00006651 (Wellik) and protocol PRO00006763 (Goldstein).

### Generation of *Hoxa11-CreER*^*T2*^ mice

Two guide sequences targeting exon 1 of *Hoxa11* were designed and cloned into the pT7-Guide Vector (Blue Heron Biotech, LLC). The guide sequence and approximate locations of both sgRNA’s, including the corresponding PAM sequence, are illustrated in Supplementary Fig. 2a. MEGAshortscript T7 kit (Life Technologies) was used to generate in vitro transcribed sgRNA’s from the pT7-Guide Vector and products were subsequently purified using the MEGAclear kit (Life Technologies). Using the pT7-Cas9-Nuclease vector (gift from Dr. Moises Mallo), Cas9 mRNA was in vitro transcribed using the mMESSAGE mMACHINE T7 ULTRA kit (Life Technologies) and purified using the MEGAclear kit (Life Technologies).

Homologous sequences flanking exon 1 of *Hoxa11* were synthesized by Blue Heron Biotech, LLC into the pUCminusMCS vector as a continuous insert separated by sequence containing restriction sites for EcoRI, NotI, and HindIII to allow for sub-cloning of *CreER*^*T2*^ and rabbit β-globin poly-adenylation signal. The 5′ homology arm contained 1.3 kb immediately upstream of the endogenous *Hoxa11* start site and 3′ homology arm contained 1.3 kb of sequence immediately downstream of sgRNA 2 as illustrated in Supplementary Fig. 2a. Sequence for CreERT2 and rabbit β-globin poly(A) signal was sub-cloned from pCAG-CreERT2 vector (gift from Connie Cepko, Addgene plasmid #14797^21^). Targeting of CreERT2 to *Hoxa11* locus preserved endogenous upstream and downstream sequence and creates a null allele; expressing CreERT2 in place of Hoxa11.

Zygote injections were performed as previously described with minor modifications^36^. C57BL/6 female mice were superovulated and mated with C57Bl/6 male mice and one-cell stage embryos were collected for microinjection. CRISPR reagents were microinjected at the following concentrations: Cas9 mRNA (100 ng/μL), each sgRNA (50 ng/μL), and targeting plasmid (20 ng/μL). Freshly injected eggs were transferred into pseudopregnant females and resulting progeny were initially screened for potential *CreER*^*T2*^ insertion via PCR. The following internal primers for CreER sequence were used: Cre Fwd: 5′ GGACATGTTCAGGGATCGCCAGGC 3′, Cre Rev: 5′ CGACGATGAAGCATGTTTAGCTG 3′. Approximate location of primers indicated in Supplementary Fig. 2a. Cre-positive animals by PCR were analyzed by Southern Blotting to confirm targeting using 5′ and 3′ flanking probes and a Cre internal probe with Nco1-digested DNA. Approximate locations of probes are illustrated in Supplementary Fig. 2a. The 453 bp 5′ probe was generated using primers: 5′ probe Fwd: 5′ TTTCGGTTCTCCTAGACGCC 3′ and 5’ probe Rv: 5′ CACGGCGTTTGCATGAGATT 3′, the 533 bp 3′ probe was generated using primers: 3′ probe Fwd: 5′ TCTGTAGTGAGCGCCTTTGG 3′ and 3′ probe Rv: 5′ GAGGTTCCCGAGAGACTCCT 3′, and the 408 bp Cre probe was generated using primers: Cre probe Fwd: 5′ GCATTACCGGTCGATGCAACGAGTGATGAG 3′ and Cre probe Rv: 5′ GAGTGAACGAACCTGGTCGAAATCAGTGCG 3′, that were randomly labeled with ^32^P-dCTP. Four animals were CreER positive via PCR and three animals showed correct targeting via Southern Blot (Supplementary Fig. 2b, c). Animal #27 displayed germline transmission of the allele. Animals from this founder were used in all subsequent experiments.

### Tamoxifen treatment

For embryonic induction, *Hoxa11-CreER*^*T2*^ or *Osx-CreERT2* male mice were mated to *Rosa*^*tdTomato/tdTomato*^ or *Hoxa11eGFP;ROSA*^*tdTomato/tdTomato*^ female mice and the vaginal plug was checked every morning. Pregnant mice received 2 mg of tamoxifen (Sigma T5648) and 1 mg/mL progesterone (Sigma P0130) dissolved in corn oil intraperitoneally at indicated embryonic day. For postnatal induction, pups of the genotype indicated in figures received 0.25 mg of tamoxifen intragastrically at P3. At least three embryos, pups, or adult animals of the indicated genotypes were examined at time points shown in figures.

### Ulnar fracture

Following procedure previously described in detail^5^. Tamoxifen was administered as described above, and animals were aged to adult stages (8–10 weeks or 10 months). Briefly, animals were anesthetized with isoflurane during procedure and provided buprenorphine preoperatively and postoperatively and carprofen during recovery period. A small incision was made along the posterior ulnar surface and the bone was exposed via blunt dissection. Using fine wire cutters, the ulna was cut at the mid-shaft. Skin was closed using sutures. Animals were sacrificed for analysis at 10 days post injury. At least three animals from each tamoxifen induction time point were examined.

### Immunohistochemistry

Limb skeletal tissues were collected at the indicated ages or time point following fracture injury. All specimens were dissected in PBS on ice and skin was removed prior to fixation for postnatal and adult tissues. Samples were fixed in 4% paraformaldehyde in PBS (embryo: 1–3 h, postnatal (P3-P7): 4–6 h, adult (2 weeks+): 1–2 days) rocking at 4 °C. Postnatal and adult tissues were decalcified in 14% EDTA for 1–7 days depending on age. Samples were equilibrated in 30% sucrose overnight prior to embedding into optimal cutting temperature medium. Cryosections were collected at 12–30 μm through indicated segments of the limb or fracture callus using the Kawamoto tape method^37^.

Immunohistochemical staining was performed using standard methods. Sections were blocked with donkey serum and incubated with primary antibodies overnight at 4 °C against Sox9 (Millipore, AB5535, 1:500), Osterix (Abcam, ab22552, 1:300), Periostin (R&D Systems, AF2955, 1:400) and Perilipin (Sigma, P1873, 1:100). Secondary antibodies were incubated at room temperature for 2 h: donkey-anti-rabbit-Alexa Fluor 647 (Thermo Fisher, A31573, 1:1000) and donkey-anti-rabbit-Alexa Fluor 488 (Thermo Fisher, A21206, 1:1000). A modified signal amplification protocol was used to visualize SOST. Following blocking, primary antibody against SOST (R&D Systems, AF1589, 1:100) was incubated overnight at 4 °C followed by donkey-anti-goat-biotin secondary (Jackson ImmunoResearch, 705-067-003, 1:400). The biotinylated secondary was detected using Vectastain Elite ABC kit (Vector Laboratories, PK-6100) and signal was amplified by Alexa Fluor 488 Tyramide reagent (Thermo Fisher, B40853). To minimize imaging complications from autofluorescence in postnatal and adult tissues, Hoxa11eGFP was visualized using chicken-anti-GFP (Abcam, ab13970, 1:2000) and donkey-anti-chicken-Alexa Fluor 488 (Invitrogen, A11039, 1:1000) in combination with aforementioned antibodies. tdTomato expression was dim at 24–72 h post-tamoxifen induction and was visualized using rabbit-anti-RFP (Rockland, 600401379, 1:1000) and donkey-anti-rabbit-Alexa Fluor 555 (Invitrogen, A31572, 1:1000). Following longer chases, tdTomato was imaged directly without the use of an antibody.

Fluorescent images were captured on an Olympus BX-51 microscope with an Olympus DP-70 camera or Leica Upright Sp5x 2-photon confocal microscope. Confocal z-stacks were obtained through entire sections at a thickness of 2 μm and images were stacked using ImageJ software. When applicable, 10× images were stitched together using Photoshop software to obtain images of entire limbs and fracture calluses.

### Flow cytometry analysis

Bone marrow cells were harvested by flushing the marrow cavity with digestion buffer (2 mg/mL collagenase IV and 3 mg/mL dispase in 1X PBS) using a 30 G needle for both the radius and ulna. To obtain bone-adherent cells, the remaining bone following bone marrow flushing was minced in digestion buffer and subjected to subsequent digestion. The digestion of all samples was carried out at 37 °C with three rounds of agitation to achieve a single cell suspension. After each cycle of digestion/agitation, cells in suspension were collected into media (DMEM, 10% calf serum) and kept at 37 °C until the entire digestion protocol was finished. Red blood cells were lysed on ice using lysis buffer at a final concentration of 1X. For staining, cells were resuspended in staining buffer (1X PBS, 0.5% BSA, 2 mM EDTA) at a concentration of 1 × 10^6^ cells/30 μL in a solution containing the following antibodies. For hematopoietic exclusion: CD45-AF700 (eBioscience, clone 30-F11, 1:100) and TER119-APC-Cy7 (Becton Dickinson, clone TER119, 1:100). For endothelial cell exclusion: CD31-PerCPCy5.5 (Becton Dickinson, clone MEC13.3, 1:100). For MSC identification: PDGFRα/CD140a-APC (eBioscence, clone APA5, 1:100) and biotinylated rat-anti-CD51 (Biolegend, clone RMV-7, 1:100) or biotinylated goat-anti-leptin receptor (R&D, BAF497, 1:50) and streptavidin-Brilliant Violet 605 (Biolegend, 405229, 1:500). For mSSC identification: CD90.1-Brilliant Violet 510 (Biolegend, clone OX-7, 1:100), CD90.2-Brilliant Violet 510 (Biolegend, clone 53-2.1, 1:100), Ly51-PerCPCy5.5 (Biolegend, clone 6C3, 1:100), CD200-PE (Biolegend, clone OX-90, 1:100), CD202b-APC (Biolegend, clone TEK4, 1:100). Following staining on ice, all samples were washed twice with staining buffer and resuspended in staining buffer containing DAPI (1:10,000) for analysis. All analyses were carried out on an LSRII Fortessa flow cytometer (BD) and results were analyzed with FlowJo (v10.2) software. Gating strategy outlined in Supplementary Fig. 1a, c. Results are presented as mean values ± standard error of the mean (SEM). No statistical method was used to predetermine sample size. Sample size was determined on the basis of previous literature and our previous experiments to give sufficient standard error of the mean, and feasible generation of experimental animals. *N*-values represent number of animals in each analyses. The experiments were not randomized and investigators were not blinded during experiments and assessment of results.

### Reporting summary

Further information on research design is available in the Nature Research Reporting Summary linked to this article.

## Supplementary information

Supplementary Information

Reporting Summary

## Data Availability

The data that support the findings of this study are available from the corresponding author upon reasonable request
