# Supplementary material for: Hox11 expressing regional skeletal stem cells are progenitors for osteoblasts, chondrocytes and adipocytes throughout life
Source: Nat Commun. 2019 Jul 18;10:3168. doi: 10.1038/s41467-019-11100-4 (PMC6639390; doi:10.1038/s41467-019-11100-4)
Supplement: Supplementary file 1 — Supplementary Information [file 41467_2019_11100_MOESM1_ESM.pdf]

# Hox11 expressing regional skeletal stem cells are progenitors for osteoblasts, chondrocytes and adipocytes throughout life.

Pineault, K.M., *et al.*

# Supplementary Information

## Supplementary Figures and Legends

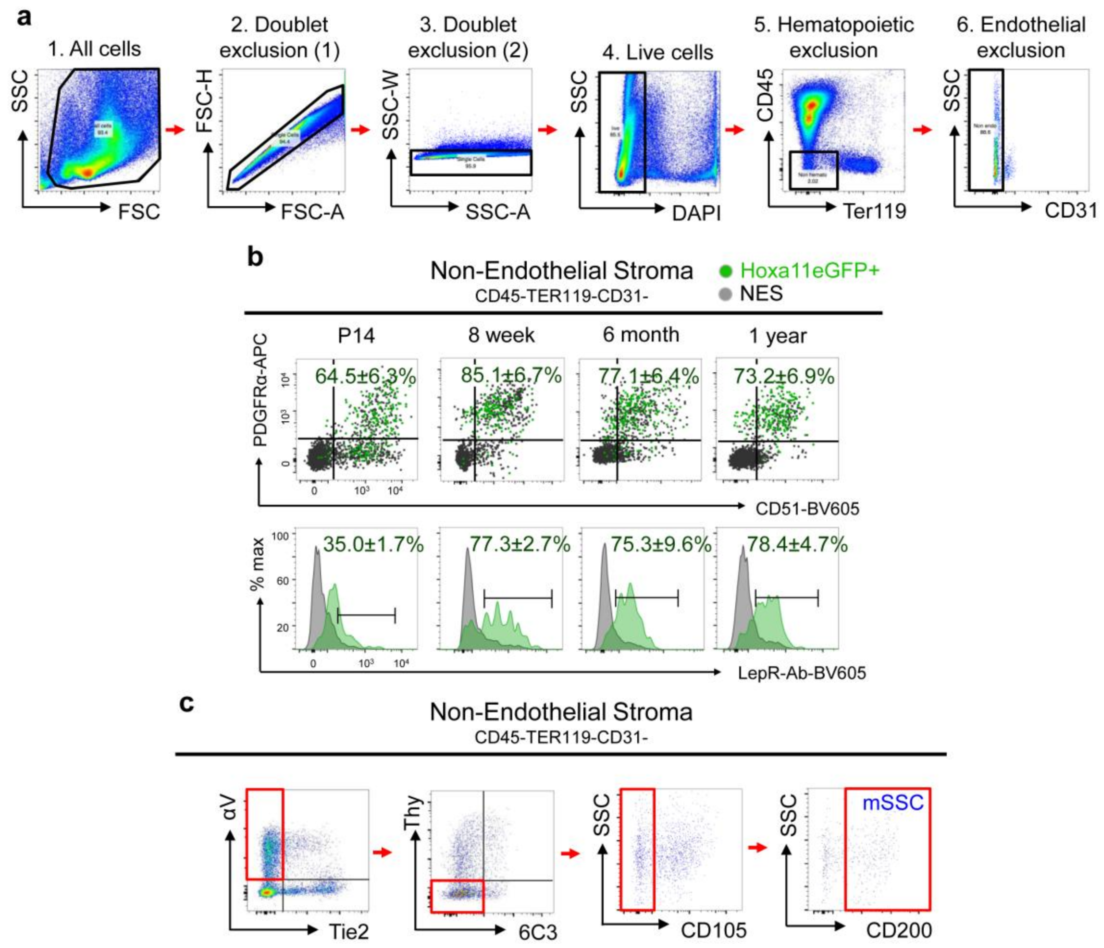


**Supplementary Figure 1: Bone adherent Hoxa11eGFP-positive cells express MSC markers PDGFRα/CD51 and Leptin Receptor.** (**a**) Gating strategy to obtain non-hematopoietic, non-endothelial stromal compartment. Example from adult (8-10wk) bone marrow. Strategy applies to data generated in Figures 2, 5, 7, 8i, and 9g and Supplementary Figures 1b, 3e, 4b, and 5. (**b**) Compliment to Figure 2a, flow cytometry analyses of bone adherent compartment (P14, 8 week, 6 month, and 1 year). Non-hematopoietic, non-endothelial stromal compartment (CD45-TER119-CD31-) was gated on PDGFRα/CD51 (top) or Leptin Receptor (LepR-Ab, bottom). Percentages reflect proportion of Hoxa11eGFP-positive population within double positive gate (top) or bracketed region of histogram (bottom). Charcoal dots or grey histogram: total non-endothelial stroma (NES), green dots or green histogram: Hoxa11eGFP-expressing non-endothelial stroma (Hoxa11eGFP+). All data presented as mean ± standard deviation. (**c**) Gating strategy to obtain mouse skeletal stem cell (mSSC) population (CD45-TER119-CD31-αV+Thy-6C3-CD105-CD200+) from non-endothelial stroma population. Strategy applies to Figure 2b-c.

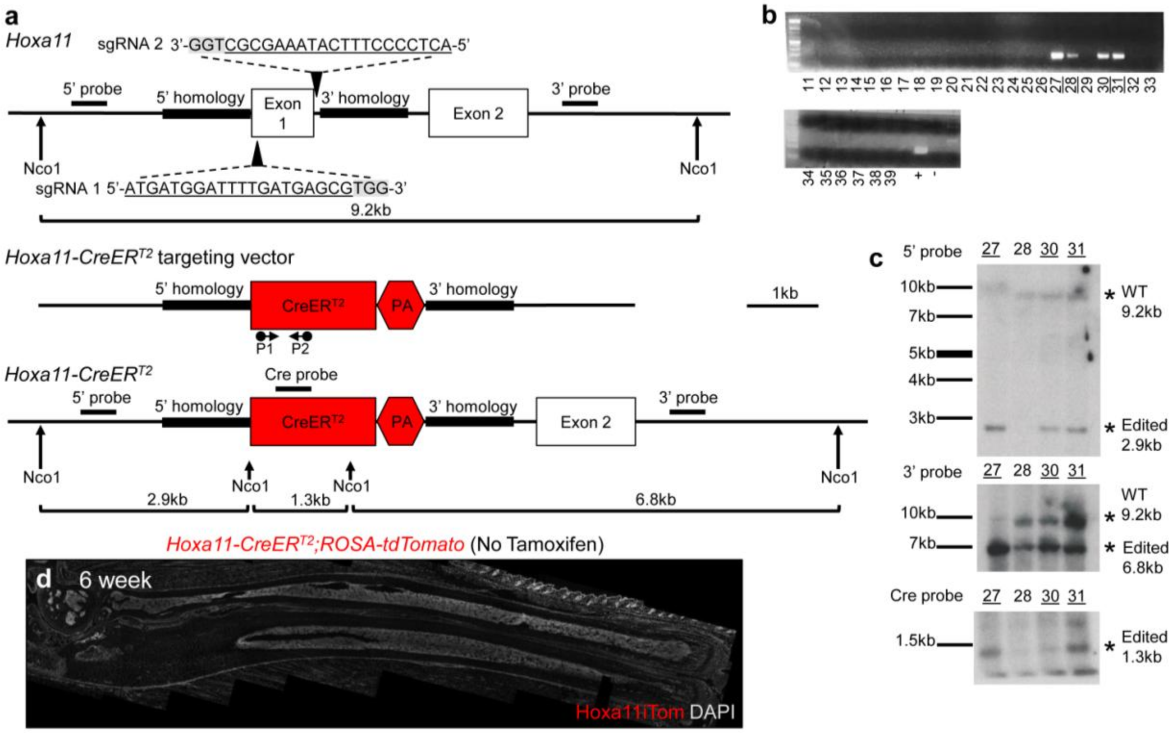


**Supplementary Figure 2: Cas9/CRISPR generation of a *Hoxa11-CreER*$^{T2}$ allele:** (**a**) Schematic of Cas9/CRISPR targeting of *Hoxa11* locus for generation of *Hoxa11-CreER*$^{T2}$ allele. Top: *Hoxa11* locus, positions and sequence of sgRNAs (grey box: PAM), Nco1 restriction sites, positions for 5' and 3' Southern Blot probes and size of wild-type (WT) fragment generated. Middle: *Hoxa11-CreER*$^{T2}$ targeting vector, 5' and 3' homology regions (thick black line), CreER$^{T2}$ and rabbit globin poly-adenylation (PA) insertion (red), and location of Cre PCR primers. Bottom: *Hoxa11-CreER*$^{T2}$ allele, Nco1 restriction sites, positions for 5', 3' and Cre Southern Blot probes and size of edited fragments generated. (**b**) PCR analysis for Cre sequence on 29 live births. (**c**) Southern Blot on four Cre-positive animals using 5' probe (top), 3' probe (middle) and Cre probe (bottom). Wild-type and edited bands and sizes as marked. (**d**) *CreER*$^{T2}$ recombination in the absence of tamoxifen in *Hoxa11-CreER*$^{T2}$*;ROSA-tdTomato* mice at 6 weeks of age. Fluorescent image - red: *Hoxa11* lineage-marked cells (Hoxa11iTom), grey: DAPI.

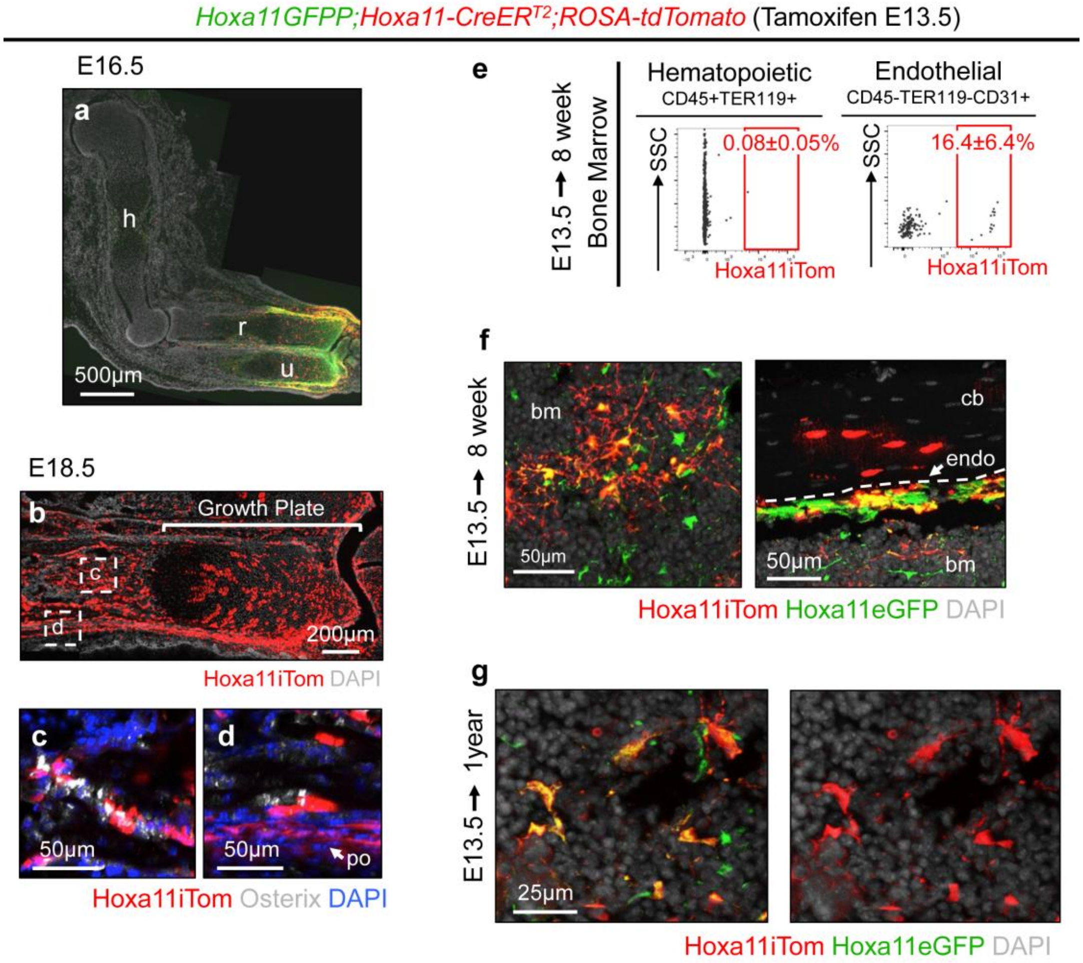


**Supplementary Figure 3: Embryonic *Hoxa11*-lineage contributes to the skeleton during development and marked stromal cells co-express Hoxa11eGFP and persist throughout life.** Pregnant dams received tamoxifen at E13.5 and resulting *Hoxa11eGFP;Hoxa11-CreER$^{T2}$;ROSA-tdTomato* mice were chased to (**a**) E16.5, (**b-d**) E18.5 (**e-f**) 8 weeks or (**g**) 1 year. (**a**) Hoxa11eGFP (green) expression and lineage contribution of *Hoxa11* lineage-marked cells (Hoxa11iTom, red) in forelimb (humerus (h), radius (r), and ulna (u)). Compliment to Figure 3f. (**b**) High magnification view of ulna growth plate. Dashed white boxes show approximate location of high magnification images (**c-d**). Co-expression of Hoxa11iTom and Osterix (white) in (**c**) primary spongiosa (**d**) periosteum (po). (**e**) Flow cytometry analyses of Hoxa11iTom cells in the hematopoietic (left, CD45+TER119+) and endothelial (right, CD45-TER119-CD31+) compartments in the bone marrow. Flow cytometry data presented as mean ± standard deviation. Percentages reflect proportion of Hoxa11iTom population within identified gate. Grey dots: total non-endothelial stroma (NES), red dots: Hoxa11iTom. (**f**) Co-expression of Hoxa11iTom and Hoxa11eGFP in the bone marrow (left) and on the bone surface (right). Cortical bone: cb, bone marrow: bm, dashed white line marks endosteal (endo) bone surface. (**g**) Hoxa11eGFP in Hoxa11iTom bone marrow stromal cells after one-year chase. All images, grey or blue: DAPI.

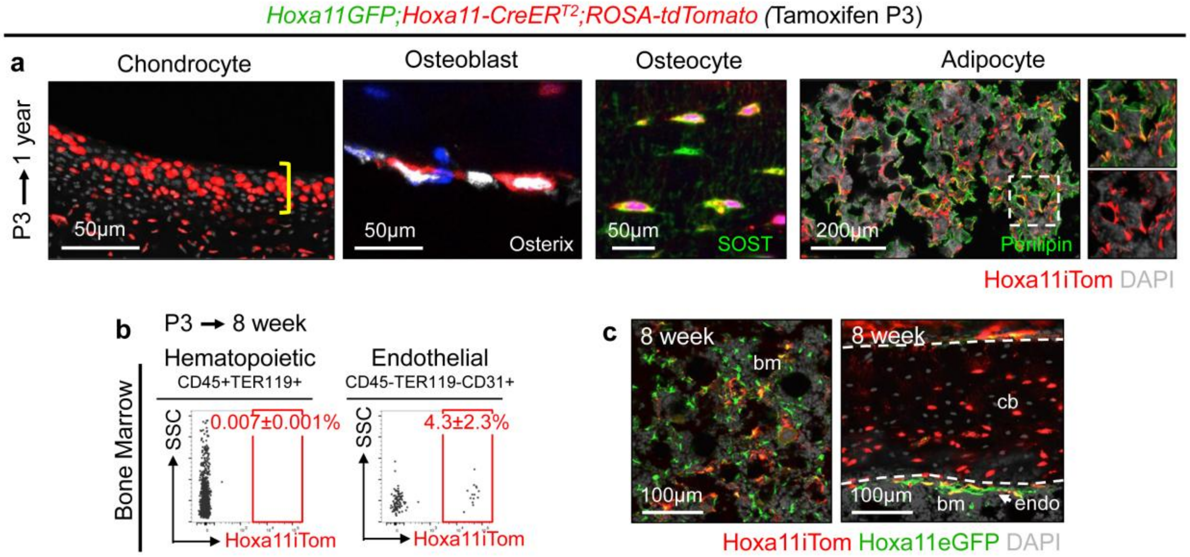


**Supplementary Figure 4: Postnatal *Hoxa11*-lineage contributes to all skeletal/mesenchymal lineages at 1 year.** P3 pups received tamoxifen and *Hoxa11eGFP;Hoxa11-CreER$^{T2}$;ROSA-tdTomato* mice were chased to (**a**) 1 year or (**b-c**) 8 weeks. (**a**) *Hoxa11* lineage-marked cells (Hoxa11iTom, red) within articular cartilage, and immunolabeling for osteoblasts (Osterix, white) on endosteal bone surface, osteocytes (SOST, green) in cortical bone, and adipocytes (Perilipin, green) in bone marrow. Articular chondrocytes marked by yellow bracket. (**b**) Flow cytometry analyses of Hoxa11iTom cells in the hematopoietic (left, CD45+TER119+) and endothelial (right, CD45-TER119-CD31+) compartments in the bone marrow. Flow cytometry data presented as mean ± standard deviation. Percentages reflect proportion of Hoxa11iTom population within identified gate. Grey dots: total non-endothelial stroma (NES), red dots: Hoxa11iTom. (**c**) Hoxa11iTom and Hoxa11eGFP (green) in bone marrow (left) and on bone surface (right). Bone marrow: bm, cortical bone: cb, white dashed lines mark periosteal and endosteal (endo) surfaces. All images, blue or grey: DAPI

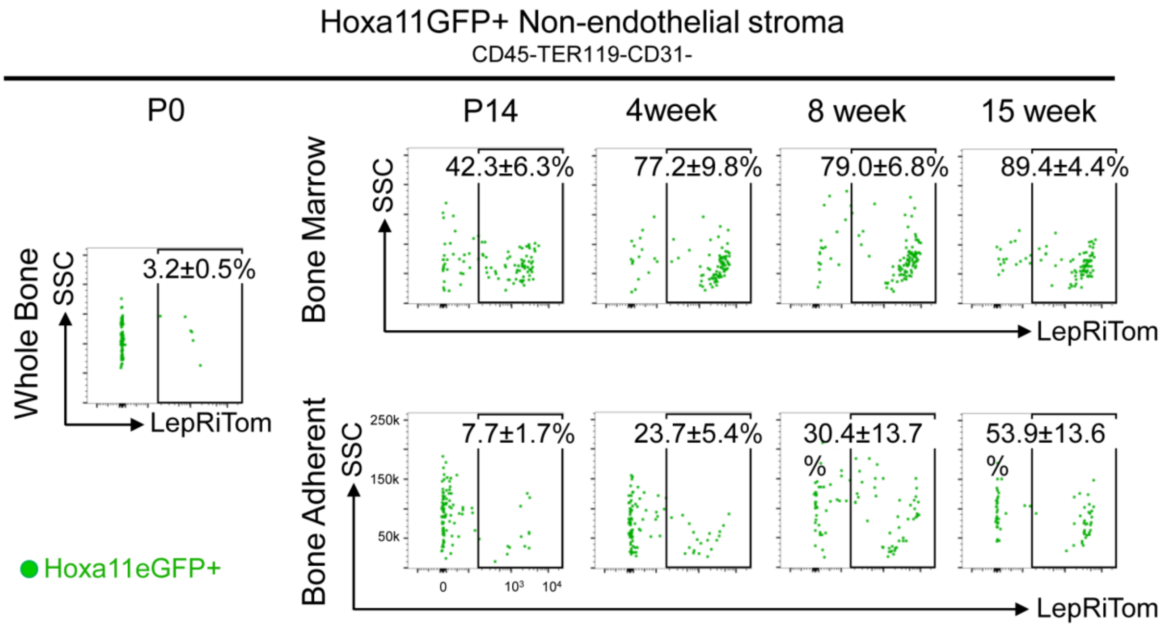


**Supplementary Figure 5: *LepR-Cre* lineage progressively overlaps with Hoxa11eGFP-positive population.** Compliment to Figure 7. Flow cytometry analyses of Hoxa11eGFP-expressing stromal cells (green) and *LepR*-Cre lineage (LepRiTom) in whole bone at P0, or bone marrow (top) and bone adherent (bottom) compartments from P14 to 15 weeks. Analysis in non-hematopoietic, non-endothelial (CD45-TER119-CD31-) compartment. Percentages reflect proportion of Hoxa11eGFP-positive population within indicated gate. All data presented as mean ± standard deviation.
