# Supplementary material for: Hox11 expressing regional skeletal stem cells are progenitors for osteoblasts, chondrocytes and adipocytes throughout life
Source: Nat Commun. 2019 Jul 18;10:3168. doi: 10.1038/s41467-019-11100-4 (PMC6639390; doi:10.1038/s41467-019-11100-4)
Supplement: Supplementary file 2 — Reporting Summary [file 41467_2019_11100_MOESM2_ESM.pdf]

nature research

Corresponding author(s): *Double-blind peer review submissions: write DBPR and your manuscript number here instead of author names.*

Last updated by author(s): *YYYY-MM-DD*

Reporting Summary

Nature Research wishes to improve the reproducibility of the work that we publish. This form provides structure for consistency and transparency in reporting. For further information on Nature Research policies, see Authors & Referees and the Editorial Policy Checklist.

Statistics

For all statistical analyses, confirm that the following items are present in the figure legend, table legend, main text, or Methods section.

n/a	Confirmed	
☐	☒	The exact sample size (n) for each experimental group/condition, given as a discrete number and unit of measurement
☐	☒	A statement on whether measurements were taken from distinct samples or whether the same sample was measured repeatedly
☒	☐	The statistical test(s) used AND whether they are one- or two-sided<br>*Only common tests should be described solely by name; describe more complex techniques in the Methods section.*
☒	☐	A description of all covariates tested
☒	☐	A description of any assumptions or corrections, such as tests of normality and adjustment for multiple comparisons
☒	☐	A full description of the statistical parameters including central tendency (e.g. means) or other basic estimates (e.g. regression coefficient) AND variation (e.g. standard deviation) or associated estimates of uncertainty (e.g. confidence intervals)
☒	☐	For null hypothesis testing, the test statistic (e.g. F, t, r) with confidence intervals, effect sizes, degrees of freedom and P value noted<br>*Give P values as exact values whenever suitable.*
☒	☐	For Bayesian analysis, information on the choice of priors and Markov chain Monte Carlo settings
☒	☐	For hierarchical and complex designs, identification of the appropriate level for tests and full reporting of outcomes
☒	☐	Estimates of effect sizes (e.g. Cohen's d, Pearson's r), indicating how they were calculated

Our web collection on statistics for biologists contains articles on many of the points above.

Software and code

Policy information about availability of computer code

Data collection	No software was used.
Data analysis	Flow cytometry data analyzed using FlowJo (v10.2).

For manuscripts utilizing custom algorithms or software that are central to the research but not yet described in published literature, software must be made available to editors/reviewers. We strongly encourage code deposition in a community repository (e.g. GitHub). See the Nature Research guidelines for submitting code & software for further information.

Data

Policy information about availability of data

All manuscripts must include a data availability statement. This statement should provide the following information, where applicable:

- Accession codes, unique identifiers, or web links for publicly available datasets
- A list of figures that have associated raw data
- A description of any restrictions on data availability

The data that support the findings of this study are available from the corresponding author upon reasonable request

Field-specific reporting

Please select the one below that is the best fit for your research. If you are not sure, read the appropriate sections before making your selection.

☒ Life sciences ☐ Behavioural & social sciences ☐ Ecological, evolutionary & environmental sciences

For a reference copy of the document with all sections, see nature.com/documents/nr-reporting-summary-flat.pdf

Life sciences study design

All studies must disclose on these points even when the disclosure is negative.

Sample size	Sample size was determined on the basis of previous literature and our previous experiments to give sufficient standard error of the mean, and feasible generation of experimental animals.
Data exclusions	Data was only excluded if the experiment was, on principle, a technical failure. Examples for exclusion include; sub-optimal antibody staining, cell death, equipment failure, and/or sample contamination.
Replication	All reasonable efforts were made to include data from two or more individual experiments to obtain sufficient sample numbers and ensure data could be replicated across experiments. These efforts included; using animals from multiple litters, distinct cohorts of tamoxifen administration, collection of flow cytometry data over different experiments on different days, and replication of immunohistochemistry staining across multiple animals.
Randomization	Mice of the indicated genotypes were randomly assigned to experiments and both male and female animals were used.
Blinding	Experiments were not blinded and investigators were aware of genotypes of experimental animals. No statistical comparisons were made between experimental groups and therefore blinding was unnecessary.

Reporting for specific materials, systems and methods

We require information from authors about some types of materials, experimental systems and methods used in many studies. Here, indicate whether each material, system or method listed is relevant to your study. If you are not sure if a list item applies to your research, read the appropriate section before selecting a response.

Materials & experimental systems

n/a	Involved in the study	
☐	☒	Antibodies
☒	☐	Eukaryotic cell lines
☒	☐	Palaeontology
☐	☒	Animals and other organisms
☒	☐	Human research participants
☒	☐	Clinical data

Methods

n/a	Involved in the study	
☒	☐	ChIP-seq
☐	☒	Flow cytometry
☒	☐	MRI-based neuroimaging

Antibodies

Antibodies used	Sox9 (Millipore, AB5535), Osterix (Abcam, ab22552), Perilipin (Sigma, P1873), donkey-anti-rabbit-Alexa Fluor 647 (Thermo Fisher, A31573), donkey-anti-rabbit-Alexa Fluor 488 (Thermo Fisher, A21206), SOST (R&D Systems, AF1589), donkey-anti-goat-biotin secondary (Jackson ImmunoResearch, 705-067-003), GFP (Abcam, ab13970), donkey-anti-chicken-Alexa Fluor 488 (Invitrogen, A11039), RFP (Rockland, 600401379), donkey-anti-rabbit-Alexa Fluor 555 (Invitrogen, A31572), CD45-AF700 (eBioscience, clone 30-F11), TER119-APC-Cy7 (Becton Dickinson, clone TER119), CD31-PerCPCy5.5 (Becton Dickinson, clone MEC13.3), PDGFRα/CD140a-APC (eBioscence, clone APA5), CD51 (Biolegend, clone RMV-7), leptin receptor (R&D, BAF497), streptavidin-Brilliant Violet 605 (Biolegend, 405229), CD90.1-Brilliant Violet 510 (Biolegend, clone OX-7), CD90.2-Brilliant Violet 510 (Biolegend, clone 53-2.1), Ly51-PerCPCy5.5 (Biolegend, clone 6C3), CD200-PE (Biolegend, clone OX-90), CD202b-APC (Biolegend, clone TEK4).
Validation	Immunohistochemistry: staining patterns were validated in our hands using "no-primary" controls. All antibodies were used to identify common cell types within the skeletal biology field and all staining was additionally compared to expected patterns and morphology accepted by the field.<br>Flow cytometry: All antibodies used have been previously validated by other labs. Relevant citations for gating strategies using these antibody combinations to identify MSC-enriched populations are reported in the text. Single antibody controls were included for every FACS experiment to ensure expected staining intensity, absence of background fluorescence, and to establish the positive/negative gate boundary.

Animals and other organisms

Policy information about studies involving animals; ARRIVE guidelines recommended for reporting animal research

Laboratory animals	All animal lines used in this study are reported in materials and methods and appropriate citations included. Generation of new Hoxa11-CreERT2 line for this study is described in detail.
Wild animals	No wild animals were used
Field-collected samples	No field collected samples were used

Ethics oversight	Ethical approval was obtained from the University of Michigan's Committee on Use and Care of Animals under protocol PRO00006651 and protocol PRO00006763.

Note that full information on the approval of the study protocol must also be provided in the manuscript.

Flow Cytometry

Plots

Confirm that:

☒ The axis labels state the marker and fluorochrome used (e.g. CD4-FITC).

☒ The axis scales are clearly visible. Include numbers along axes only for bottom left plot of group (a 'group' is an analysis of identical markers).

☒ All plots are contour plots with outliers or pseudocolor plots.

☒ A numerical value for number of cells or percentage (with statistics) is provided.

Methodology

Sample preparation	Detailed sample preparation description is provided in materials and methods. All cells were obtained from mouse skeleton and single cell suspensions were obtained as described in methods.
Instrument	All data were obtained on a LSRII Fortessa flow cytometer (BD biosciences)
Software	All results were analyzed using FlowJo (v10.2).
Cell population abundance	The cell population is rare within the bone marrow. This population exists within the non-hematopoietic, non-endothelial stromal compartment of the bone marrow (~1-2% of all cells in the bone marrow). Within the NES, Hox11-expressing stromal cells represent ~5-8% of this population (Rux et al., Dev Cell (2016)).
Gating strategy	Gating strategy is outlined in Supplemental Figure 1a. Standard doublet exclusion gating was performed using forward scatter and side scatter and live/dead exclusion was performed using DAPI. Live cells were gated on CD45 and Ter119 to obtain the double-negative (non-hematopoietic) population. Non-hematopoietic cells were further gated on CD31 to obtain the negative (non-endothelial) population. Non-hematopoietic, non-endothelial stromal cells were gated on either PDGFRa;CD51 or Leptin Receptor to identify the MSC-enriched fraction. Alternatively, non-hematopoietic, non-endothelial stromal cells were sequentially gated for Tie-negative/alpha-V-positive, Thy-negative/6C3-negative, CD105-negative, and CD200-positive to obtain the previously defined mouse skeletal stem cell (mSSC) population.

☒ Tick this box to confirm that a figure exemplifying the gating strategy is provided in the Supplementary Information.
